# Ecological Responses of *Cyanotis arachnoidea* C.B. Clarke and Its Rhizosphere Microbiome in Zinc Mine Tailings: Insights for Post-Mining Restoration

**DOI:** 10.3390/ijms27219684

**Published:** 2026-10-29

**Authors:** Nichanun Kutrasaeng, Praphat Kawicha, Aphidech Sangdee, Woranan Nakbanpote

**Affiliations:** 1 Department of Biology, Faculty of Science, Mahasarakham University, Maha Sarakham 44150, Thailand; nichakarbkesorn@gmail.com (N.K.); aphidech.s@msu.ac.th (A.S.); 2 Plant Genome and Disease Research Unit, Department of Agriculture and Resources, Faculty of Natural Resources and Agro-Industry, Kasetsart University Chalermphrakiat Sakon Nakhon Province Campus, Sakon Nakhon 47000, Thailand; praphat.kawicha@gmail.com

**Keywords:** *Cyanotis arachnoidea*, phytoremediation, zinc and cadmium stress, rhizosphere microbiome, zinc mine tailings

## Abstract

Mining activities severely disrupt soil ecosystems through heavy metal contamination. This study evaluated the phytoremediation potential and rhizosphere microbial dynamics of *Cyanotis arachnoidea* C.B. Clarke (confirmed via *rbcLa* and *trnH–psbA* barcoding), a native plant from Thai zinc mines. Pot experiments demonstrated that plants grown in tailings-contaminated soil exhibited reduced growth and distinct leaf reddening, accompanied by significantly elevated anthocyanin levels. Conversely, proline levels decreased, whereas chlorophyll content and antioxidant parameters (total phenolic content (TPC), total flavonoid content (TFC), and free radical scavenging activity (FRSA)) changed only slightly. Based on zinc (Zn) and cadmium (Cd) uptake and translocation profiles, growth characteristics, and physiology, *C. arachnoidea* was classified as a highly Zn-tolerant phytostabilizer and a high-translocating Cd excluder. Furthermore, high-throughput sequencing (HTS) and culture-dependent analyses demonstrated that metal toxicity reduced bacterial alpha diversity but selectively enriched stress-tolerant taxa—such as *Sphingomonas*, *Bacillus*, Bacteroidota, and Chloroflexi—compared with the nutrient-cycling Acidobacteriota that dominated the cover soil. Collectively, its physiological adaptability, capacity for Zn phytostabilization, effective Cd exclusion strategy, and association with a stress-resilient microbiome make *C. arachnoidea* a promising candidate for the ecological restoration of multi-metal-contaminated sites.

## 1. Introduction

Mining operations generate large quantities of metal-contaminated waste that profoundly alter soil chemistry, vegetation structure, and microbial ecosystem stability [1]. In particular, open-pit mining, land clearing, and topsoil removal have led to severe habitat degradation, biodiversity loss, and the accumulation of fine-grained tailings with hazardous trace elements. In Thailand, the Minerals Act B.E. 2560 (2017) [2] mandates the rehabilitation of former mining areas through soil amelioration, revegetation, and rigorous environmental monitoring. In this context, a large former open-pit Zn mine (3.32 km^2^) in Pha Daeng District, Tak Province, Thailand, has been undergoing restoration since 2016 with the aim of returning it to forest, with parts to be converted into a botanical garden [3]. Rehabilitating this closed open-pit Zn mine has involved backfilling the pit with uncontaminated soil (cover soil) before planting, as well as remediating the tailings piles, which still contain high levels of Zn and Cd [4]. Although Zn is an essential micronutrient involved in photosynthesis, reproductive development, and antioxidant systems, maintaining Zn within a narrow physiological threshold is critical to prevent phytotoxicity [5]. Excess Zn and Cd can disrupt cellular homeostasis; Cd, a guest metal in Zn mineralization, can lead to biomass reduction, chlorosis, root inhibition, and, in severe cases, plant death [6,7]. Establishing native plant species in these metal-enriched soils is a key component of long-term ecosystem restoration.

Native plants are essential for mining restoration due to their strong adaptability and ecological functions, contributing to both ecological restoration and socio-economic sustainability [8,9]. Additionally, native species minimize invasive risks and require fewer inputs due to their local resilience [10]. ‘Ton Sangkasi’ (literally “zinc plant”) is the local Thai name for a plant associated with zinc deposits; historically, it grew on zinc ore and had reddish leaves. Researchers previously studied this perennial herbaceous plant for its ability to accumulate Zn and Cd under the scientific name *Cyanotis tuberosa* Schult.f. [11,12]. However, based on plant distribution data and a comparison of historical photographs that revealed no tuber (storage root), its scientific name has been revised to *Cyanotis arachnoidea* C.B. Clarke (Commelinaceae) [13]. Notably, several *Cyanotis* species, such as *C. longifolia* and *C. cerifolia* [14] and *C. lanata* [15], have been reported to tolerate high metal concentrations and contribute to bioremediation. Plants cope with excess Zn and Cd through metal sequestration, transport regulation, detoxification processes, and antioxidant systems [16,17]. Exposure to heavy metals triggers the accumulation of antioxidant compounds, such as phenolics and flavonoids, which serves as a critical defense mechanism by scavenging free radicals and protecting essential cellular structures from oxidative damage [6,18]. Therefore, evaluating antioxidant responses may provide useful insights into the adaptive mechanisms of *C. arachnoidea* under metal stress.

Successfully restoring *C. arachnoidea* populations could achieve dual objectives: facilitating long-term phytoremediation and preserving indigenous biodiversity. However, *C. arachnoidea* has disappeared from the area as a result of past open-pit zinc mining. Therefore, it is necessary to reintroduce and propagate the *Cyanotis* species found in Phu Kradueng National Park, which lies in the same Thongchai mountain range as the zinc mine. Because the reintroduced material originated outside the mine, its identity was verified using morphological characteristics and DNA barcoding techniques, employing the genetic markers *rbcLa* (a region of the ribulose-1,5-bisphosphate carboxylase/oxygenase large subunit gene, *rbcL*) and *trnH–psbA* (non-coding intergenic spacer) [19,20]. However, little is known about the physiological adaptation of *C. arachnoidea* to Zn- and Cd-rich substrates and its association with rhizosphere microbial communities. The rhizosphere microbiome is a critical determinant of plant performance, promoting growth and metal stress tolerance [21,22]. High concentrations of heavy metals typically reduce microbial diversity and enrich for stress-tolerant or metal-resistant taxa that can influence metal bioavailability and soil function [23,24]. HTS based on operational taxonomic units (OTUs) or amplicon sequence variants (ASVs) provides a robust framework for elucidating rhizosphere interactions and microbial community responses to soil conditions and pollution [25,26]. Furthermore, isolating and identifying culturable bacteria from soil remains crucial [25,27], particularly for future applications of bacteria with both metal tolerance and plant growth-promoting properties [21,28].

Therefore, this research aims to (i) confirm the identity of *C. arachnoidea* using *rbcLa* and *trnH–psbA* DNA barcoding; (ii) evaluate its growth, metal accumulation, and physiological responses when grown in the cover and tailings-contaminated soils; and (iii) characterize rhizosphere bacterial communities using culture-dependent and 16S rRNA amplicon sequencing approaches. The findings will provide ecological and microbiological insights to support the restoration of native vegetation in former zinc-mining areas undergoing rehabilitation.

## 2. Results

### 2.1. Plant Identification

**Figure 1:**
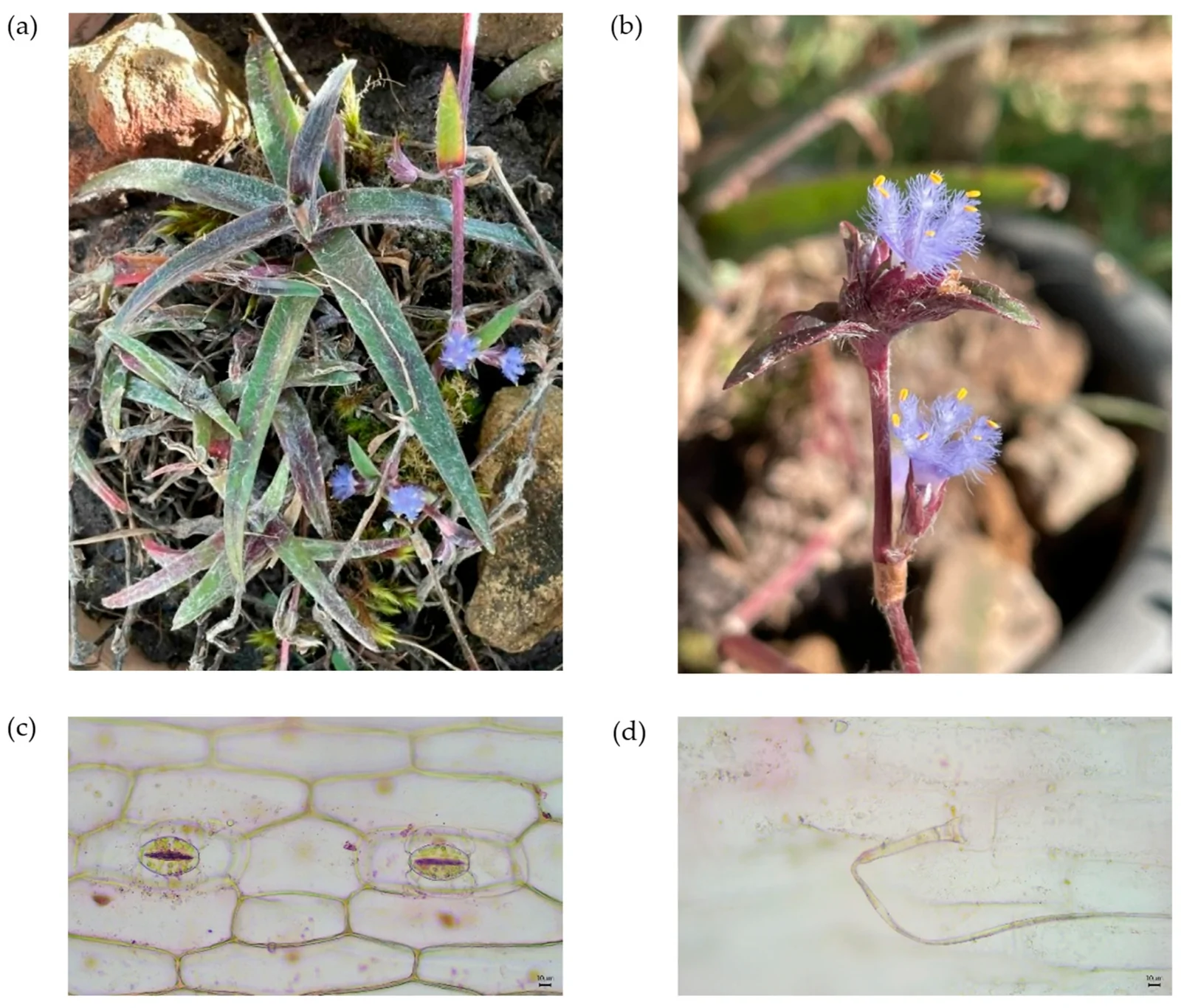
Morphological and anatomical characteristics of *Cyanotis* plants collected from Phu Kradueng National Park, Loei Province, Thailand: (**a**) basal rosette leaves, decumbent stems, linear cauline leaves, and dense arachnoid hairs; (**b**) inflorescence with bracts and bracteoles; (**c**) paracytic stomata; and (**d**) hair-like, non-glandular trichomes.

The characteristics of the plants used in this experiment are shown in Figure 1. Plants displayed elliptic-lanceolate to oblong basal rosette leaves, decumbent stems, linear cauline leaves, and dense arachnoid hairs on leaves (Figure 1a). The inflorescence was head-like, with inflorescence bracts and bracteoles (Figure 1b). Stomata were paracytic and found on both adaxial and abaxial surfaces (Figure 1c), and trichomes were hair-like and non-glandular (Figure 1d). These morphological and anatomical characteristics were consistent with the species *Cyanotis arachnoidea* C.B. Clarke in the family Commelinaceae.

**Figure 2:**
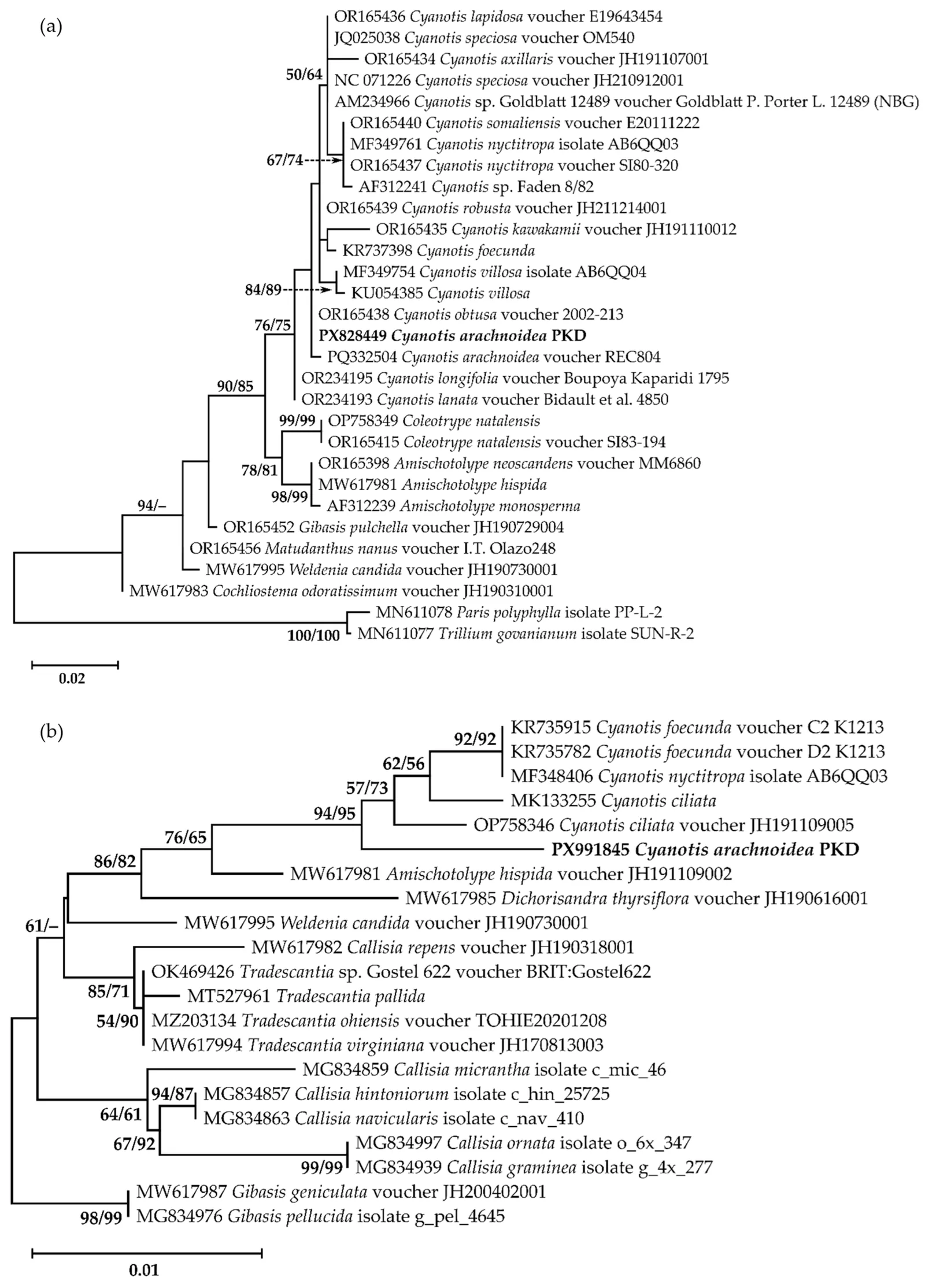
Maximum Likelihood (ML) phylogenetic trees (Tamura 3-parameter model; the tree with the highest log likelihood is shown) of *Cyanotis* plants collected from Phu Kradueng National Park (PKD) and related taxa based on chloroplast DNA sequences: (**a**) phylogeny reconstructed using the *rbcLa* sequence, and (**b**) phylogeny reconstructed using the *trnH-psbA* intergenic spacer sequence. Numbers at nodes indicate ML/NJ bootstrap support values from 1000 replicates. Scale bars represent nucleotide substitutions per position.

Two chloroplast DNA markers, *rbcLa* (567 bp) and *trnH-psbA* (705 bp), were sequenced to confirm species identity at the molecular level. In the *rbcLa* phylogenetic tree (Figure 2a), the Cyano-PKD-rbcLa sequence clustered tightly within the *Cyanotis* clade. It formed a distinct monophyletic subclade with *Cyanotis obtusa* and *Cyanotis arachnoidea*, with ML/NJ bootstrap support values of 90/85%, indicating a close evolutionary relationship with these reference species. In the *trnH-psbA* phylogenetic tree (Figure 2b), the intergenic spacer sequence (Cyano-PKD-trnH-psbA) resolved as a well-supported sister lineage (76/65% ML/NJ bootstrap) to a robust core clade comprising other known *Cyanotis* species. Outgroup taxa from related genera within Commelinaceae formed distinct basal lineages. The combined morphological and molecular evidence supports the identification of the plant collected from Phu Kradueng National Park (PKD) as *Cyanotis arachnoidea*.

### 2.2. Plant Growth and Metal Accumulation

#### 2.2.1. Comparative Analysis of Soil Properties

The initial soil data (day 0) (Table 1) showed that total Zn and Cd contents in the cover soil were 17.6- and 8.5-fold lower, respectively, than those of the tailings-contaminated soil. In addition, the cover soil excavated from an uncontaminated area in Tak Province showed lower mineral content (Fe, P, and K) and lower C and N levels than the tailings-contaminated soil in the mine. After 60 days of cultivation, total Zn and Cd had decreased significantly in all four soils, whereas extractable Zn had increased. The initial pH of the cover soil was higher than that of the tailings-contaminated soil; after pot cultivation, pH decreased and C and N levels increased in all soils. The marked decreases in available P and K and in total Fe across all four soil types, which were most pronounced in the tailings-contaminated soil, might be partly attributable to plant uptake.

#### 2.2.2. Plant Growth and Physiological Responses

**Figure 3:**
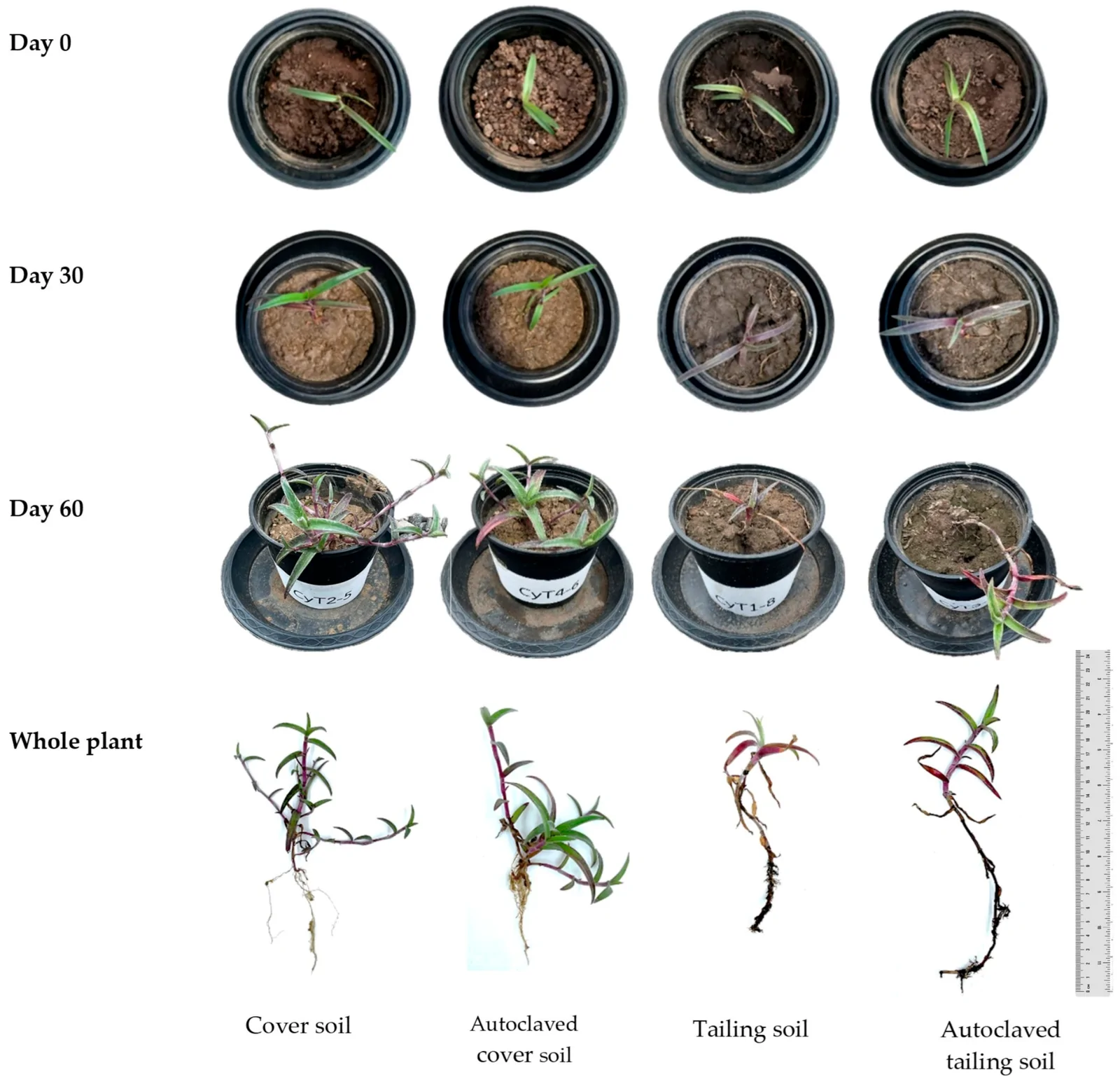
Morphological appearances of *Cyanotis arachnoidea* grown in untreated and autoclaved cover and tailings-contaminated soils. The images show pot-grown plants at day 0, day 30, and day 60, along with the entire plant structures (shoot and root systems) harvested at day 60.

**Figure 4:**
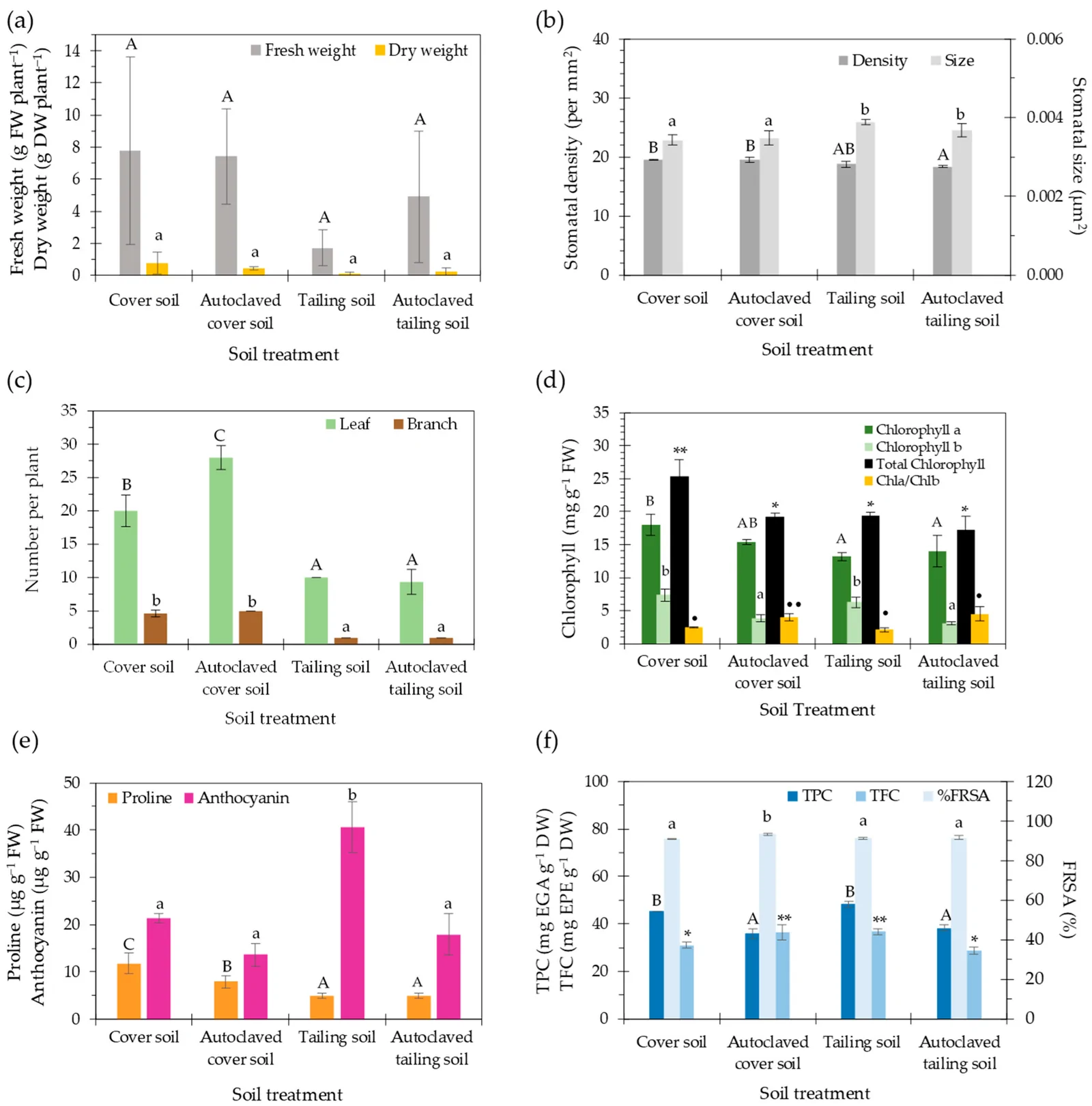
Growth and physiological responses of *Cyanotis arachnoidea* in untreated and autoclaved cover and tailings-contaminated soils after 60 days: (**a**) leaf and branch numbers, (**b**) stomatal size and density, (**c**) fresh and dry weights, (**d**) chlorophyll content and chlorophyll *a*/*b* ratio, (**e**) proline and anthocyanin contents, and (**f**) TPC, TFC, and FRSA. Data are presented as means ± SD (*n* = 3). Different letters (A–C, a–b), asterisks (* and **), and dots (• and ••) indicate significant differences among treatments (*p* < 0.05) for their respective parameters.

*C. arachnoidea* grown in tailings-contaminated soils exhibited visible metal stress symptoms. As shown in Figure 3, plant leaves in both untreated and autoclaved tailings-contaminated soils turned reddish-purple after 30 days of planting. After 60 days, plants grown in the tailings-contaminated soil showed marked morphological differences from those grown in the cover soils. Plants grown in the cover soils produced 20 to 28 leaves per plant, whereas leaf numbers in the tailings-contaminated soils were restricted to approximately 10 leaves per plant. Branch proliferation was observed only in the cover soils (Figure 4a). Plants grown in the tailings-contaminated soils had significantly larger stomata, whereas stomatal density showed slight but statistically significant variations among the treatments (Figure 4b). Although large standard deviations resulted in no significant differences in fresh and dry plant weights, plants grown in the tailings-contaminated soils showed a trend toward lower mean values (Figure 4c). Total chlorophyll content was significantly higher in plants grown in the untreated cover soil than in those in all other treatments (Figure 4d). Furthermore, autoclaving resulted in a significant decrease in chlorophyll *b* content in both soil types, whereas chlorophyll *a* content remained relatively stable, leading to an increase in the chlorophyll *a*/*b* ratio (Figure 4d). Anthocyanin content increased markedly in plants grown in the untreated tailings-contaminated soil, whereas proline content significantly declined in the tailings-contaminated soils compared with the cover soils (Figure 4e). TPC and TFC varied significantly across the soil treatments (Figure 4f), where autoclaving generally reduced TPC levels. Meanwhile, FRSA remained consistently high (>90%) across all treatments, despite minor statistically significant differences.

**Figure 5:**
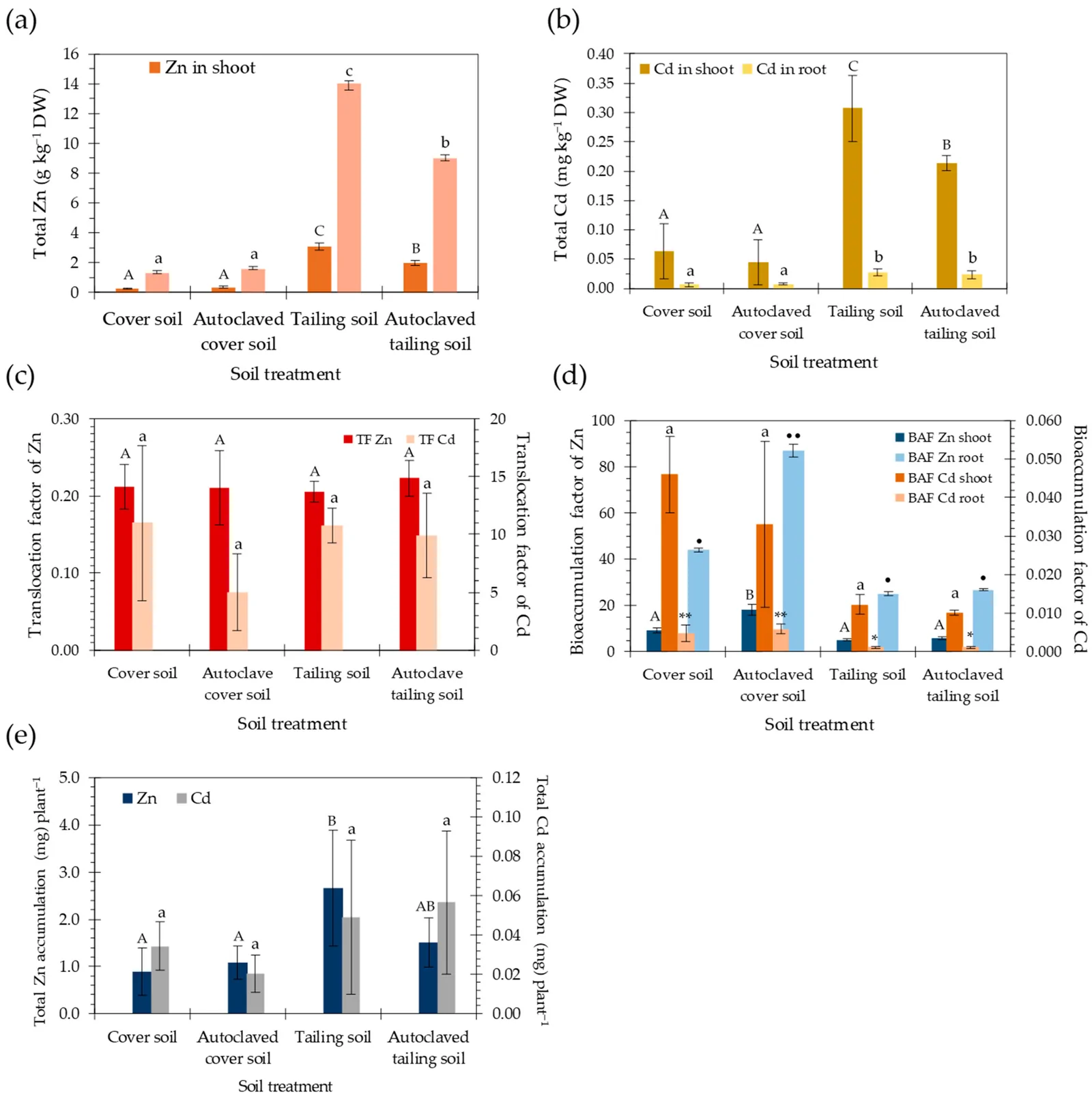
Zn and Cd uptake and transport profiles in *Cyanotis arachnoidea* grown in untreated and autoclaved cover and tailings-contaminated soils for 60 days: (**a**) total Zn concentration in shoots and roots, (**b**) total Cd concentration in shoots and roots, (**c**) translocation factors (TF), (**d**) bioaccumulation factors (BAF), and (**e**) total net accumulation of Zn and Cd per plant. Data are presented as means ± SD (*n* = 3). Different letters (A–C, a–c), asterisks (* and **), and dots (• and ••)indicate significant differences among treatments (*p* < 0.05) for their respective parameters.

Figure 5a,b show the Zn and Cd concentrations, respectively, in the shoot and root tissues of the plants. The results demonstrated that plants grown in the tailings-contaminated soils had significantly higher Zn and Cd concentrations than those grown in the cover soils. In particular, Zn concentration was higher in the roots (14.02 g kg^−1^ DW) than in the shoots (3.07 g kg^−1^ DW), whereas Cd concentration was higher in the shoots (0.31 mg kg^−1^ DW) than in the roots (0.03 mg kg^−1^ DW). Furthermore, autoclaving the tailings-contaminated soil significantly reduced the accumulation of both metals in plant tissues. The translocation factor (TF) for Cd, calculated as the ratio of shoot to root metal concentrations, was markedly high in the tailings-contaminated soils (9.1–10.6), whereas the TF for Zn remained consistently low across all treatments (0.20–0.22) (Figure 5c). A TF value > 1 indicates that *C. arachnoidea* efficiently translocated Cd from roots to shoots, even when roots accumulated high levels of Zn. Figure 5d shows the bioaccumulation factors (BAF) for both shoots (BAF_shoot_) and roots (BAF_root_), calculated as the ratio of metal concentrations in plant tissues to available metal concentrations in the soil. The significantly higher BAF_shoot_ and BAF_root_ values for both Zn and Cd observed in plants grown in the cover soils compared with the tailings-contaminated soils may be attributed to concentration-dependent bioaccumulation, whereby BAF decreases as soil metal concentration increases. All BAF_shoot_ and BAF_root_ values for Cd remained below 1 (<0.05), indicating a low potential for Cd bioaccumulation from soil into plant tissues, whereas those for Zn were substantially greater than 1 (ranging from 5 to 87).

Partial microbial suppression, demonstrated using autoclaved cover and tailings-contaminated soils, suggested a role for the native rhizosphere microbiome in plant physiology and phytoremediation. In the tailings-contaminated soils, Zn and Cd accumulation in plant tissues decreased significantly after autoclaving (Figure 5a,b). This might be attributed to a reduction in soil microorganisms with metal-solubilizing properties, resulting in lower levels of available Zn and Cd (Table 1). The differing levels of Zn and Cd accumulated in plant tissues between plants grown in the autoclaved tailings-contaminated soil and those in the untreated tailings-contaminated soil (Figure 5a,b) corresponded to differences in anthocyanin content in the leaves (Figure 4e). In addition to the notably altered anthocyanin, the slight reduction in chlorophyll *b* content (Figure 4d), proline (Figure 4e), and TPC (Figure 4f) in plants grown in the autoclaved soils might be due to partial microbial suppression, as indicated by the lower bacterial counts in the autoclaved bulk soils at day 0 (Table 2).

### 2.3. Evaluation of Rhizosphere Bacterial Diversity and Community

To investigate potential microbial contributions to plant adaptation under metal-rich conditions, rhizosphere bacterial communities associated with *C. arachnoidea* grown in the untreated cover and tailings-contaminated soils were analyzed using culture-dependent and culture-independent methods.

#### 2.3.1. Bacterial Isolation and Identification

Although the tailings-contaminated soil had higher levels of organic carbon and total nitrogen than the cover soil (Table 1), the bulk cover soil had a higher number of bacterial colonies (Table 2). The initial number of bacteria in the soil was reduced by autoclaving, but not all were eliminated (Table 2). Residual bacterial colonies detected after autoclaving may have originated from heat-tolerant or spore-forming microbial populations. The number of colonies in the rhizosphere of plants grown for 60 days increased compared with that in the initial bulk soil (Day 0). The Shannon-Wiener index (*H*) of bacteria from the tailings-contaminated soil tended to be lower than that from the cover soil. Species richness (*S*) indices in both autoclaved soils were slightly lower than those in the corresponding untreated soils, whereas evenness (*E_H_*) showed no significant difference.

**Figure 6:**
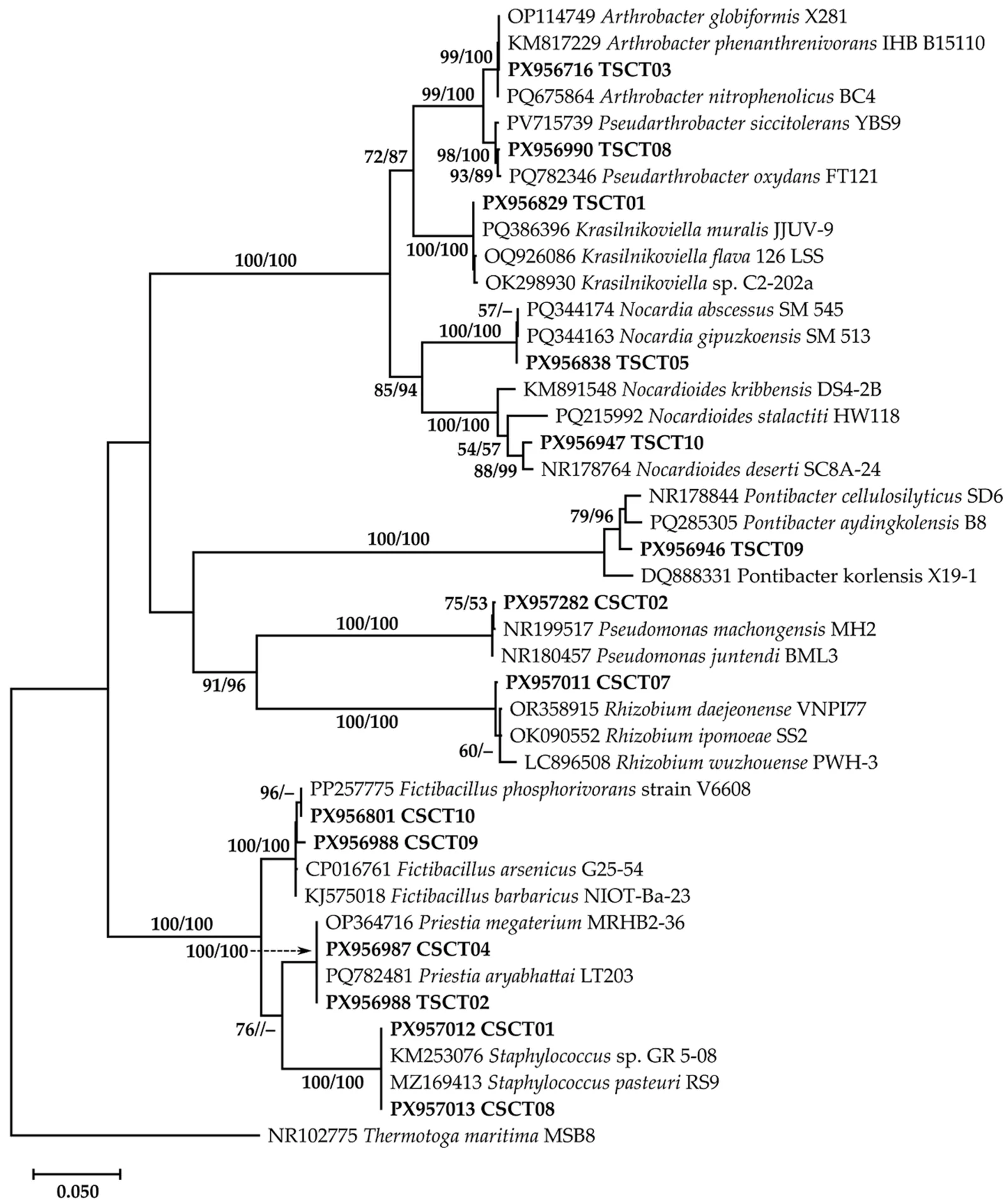
Phylogenetic analysis of 16S rRNA gene sequences of 14 culturable bacteria isolated from the rhizosphere of *Cyanotis arachnoidea* grown in untreated cover and tailings-contaminated soils, and NCBI reference taxa. The tree topology was constructed using Maximum Likelihood (ML) under the Kimura 2-Parameter model with Gamma distribution and Invariant Sites (K2+G+I). Numbers at nodes represent ML/NJ bootstrap support values (>50%) from 1000 replicates (‘–’ indicates <50% in NJ). Scale bar indicates 0.050 substitutions per nucleotide position.

Fourteen bacterial isolates exhibiting distinct colony morphologies and Gram-staining characteristics (Table S1) were obtained from the rhizosphere of *C. arachnoidea* plants grown in the untreated soils and subsequently identified by 16S rRNA gene sequencing. Isolates were coded according to their source with the prefixes CS (cover soil) or TS (tailings-contaminated soil), followed by CT (*Cyanotis*) and a sequential isolation number. The phylogenetic tree (Figure 6) shows that the bacterial isolates were distributed among four major phyla: Firmicutes (6 isolates), Proteobacteria (2 isolates), Actinobacteria (5 isolates), and Bacteroidetes (1 isolate). Most TSCT isolates were affiliated with Actinobacteria and Bacteroidetes, while CSCT isolates predominantly belonged to Firmicutes and Proteobacteria. Isolates CSCT09 and CSCT10 were closely related to *Fictibacillus* sp., while CSCT04 and TSCT02 were identified as *Priestia* sp. Furthermore, CSCT01 and CSCT08 belonged to the genus *Staphylococcus*, and CSCT07 was closely related to *Rhizobium* sp. Among the various bacterial isolates, TSCT01, TSCT03, TSCT05, TSCT08, TSCT09, and TSCT10 belonged to *Krasilnikoviella* sp., *Arthrobacter* sp., *Nocardia* sp., *Pseudarthrobacter* sp., *Pontibacter* sp., and *Nocardioides* sp., respectively. The culture-dependent results provided initial insight into the possible effects of Zn and Cd on the rhizosphere bacteria of *C. arachnoidea*. To provide a more comprehensive view of rhizosphere bacterial communities, 16S rRNA amplicon sequencing was subsequently performed.

#### 2.3.2. High-Throughput Profiling of Rhizosphere Bacterial Communities

**Figure 7:**
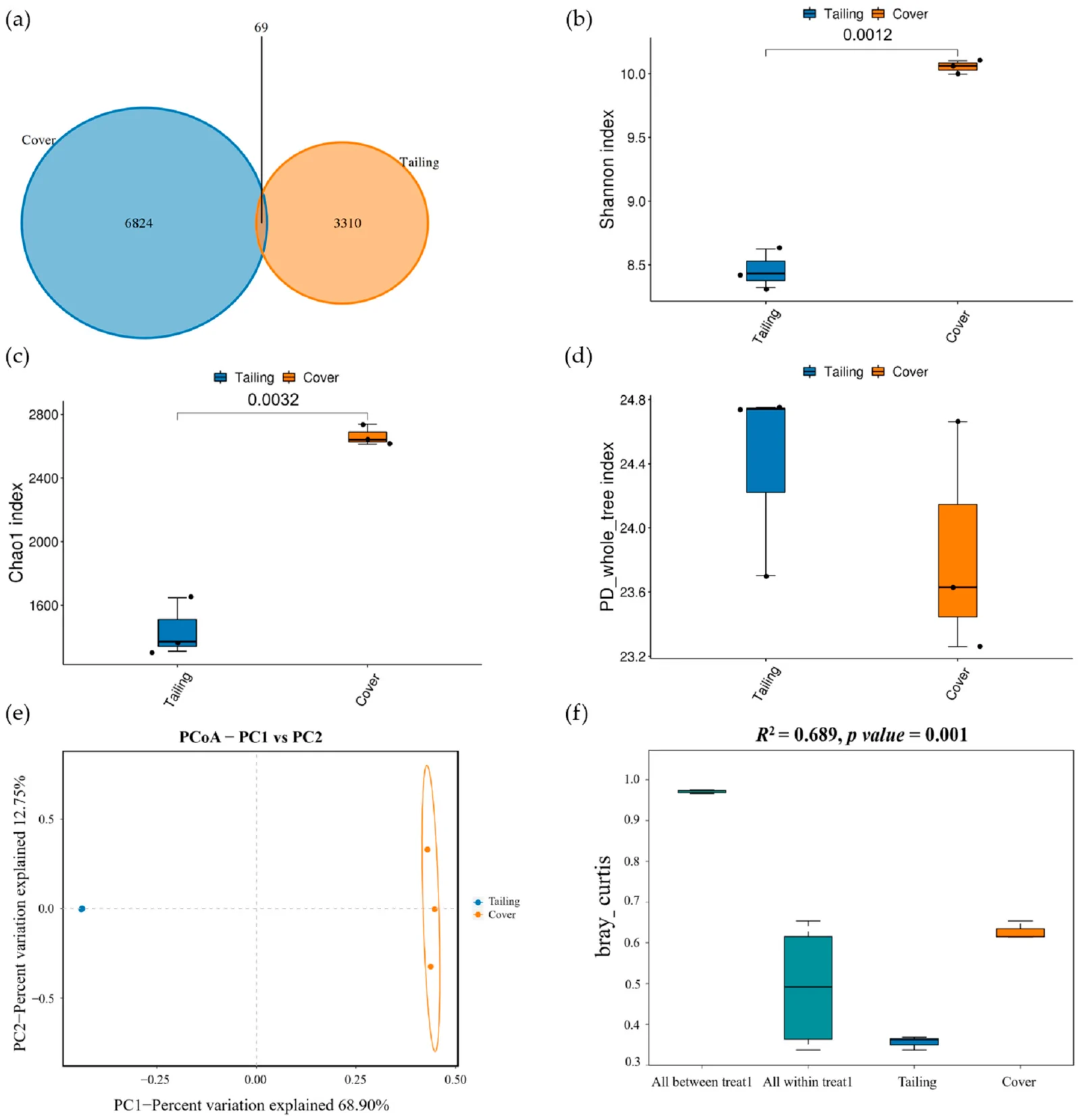
Microbial community analysis of the rhizosphere of *Cyanotis arachnoidea* grown in untreated cover and tailings-contaminated soils: (**a**) Venn diagram of shared and unique ASVs; (**b**–**d**) Alpha diversity indices (Shannon, Chao1, and PD whole tree), (**e**) PCoA plot based on Bray-Curtis distance, and (**f**) PERMANOVA analysis showing dissimilarity within and between groups.

A total of 305,879 high-quality sequences were obtained from the rhizosphere of *C. arachnoidea* plants and clustered into 10,203 amplicon sequence variants (ASVs). Rhizosphere bacterial communities differed markedly between the cover soil and the tailings-contaminated soil. The cover soil exhibited substantially higher bacterial richness (6824 unique ASVs) than the tailings-contaminated soil (3310 unique ASVs). Only 69 ASVs were shared between the two soil types, indicating strong shifts in community composition under metal-rich conditions (Figure 7a).

Alpha diversity indices, including the Shannon index (Figure 7b) and the Chao1 index (Figure 7c), indicated significantly higher bacterial richness and evenness in the cover soil than in the tailings-contaminated soil. In contrast, the phylogenetic diversity (PD) index (Figure 7d) showed higher phylogenetic diversity in the tailings-contaminated soil. Beta diversity analysis via PCoA (Bray-Curtis) (Figure 7e) confirmed a clear separation between the two groups along the first principal coordinate (PC1), which explained 68.9% of the total variation. PERMANOVA (Figure 7f) further validated these findings, with a high effect size (R^2^ = 0.689) and *p* = 0.001. The dissimilarity between groups was markedly higher than within groups. Preliminary correlation analysis (Table S2) indicated potential associations between the chemical properties of both bulk soils after 60 days of plant growth (Table 1) and microbial biodiversity indices.

**Figure 8:**
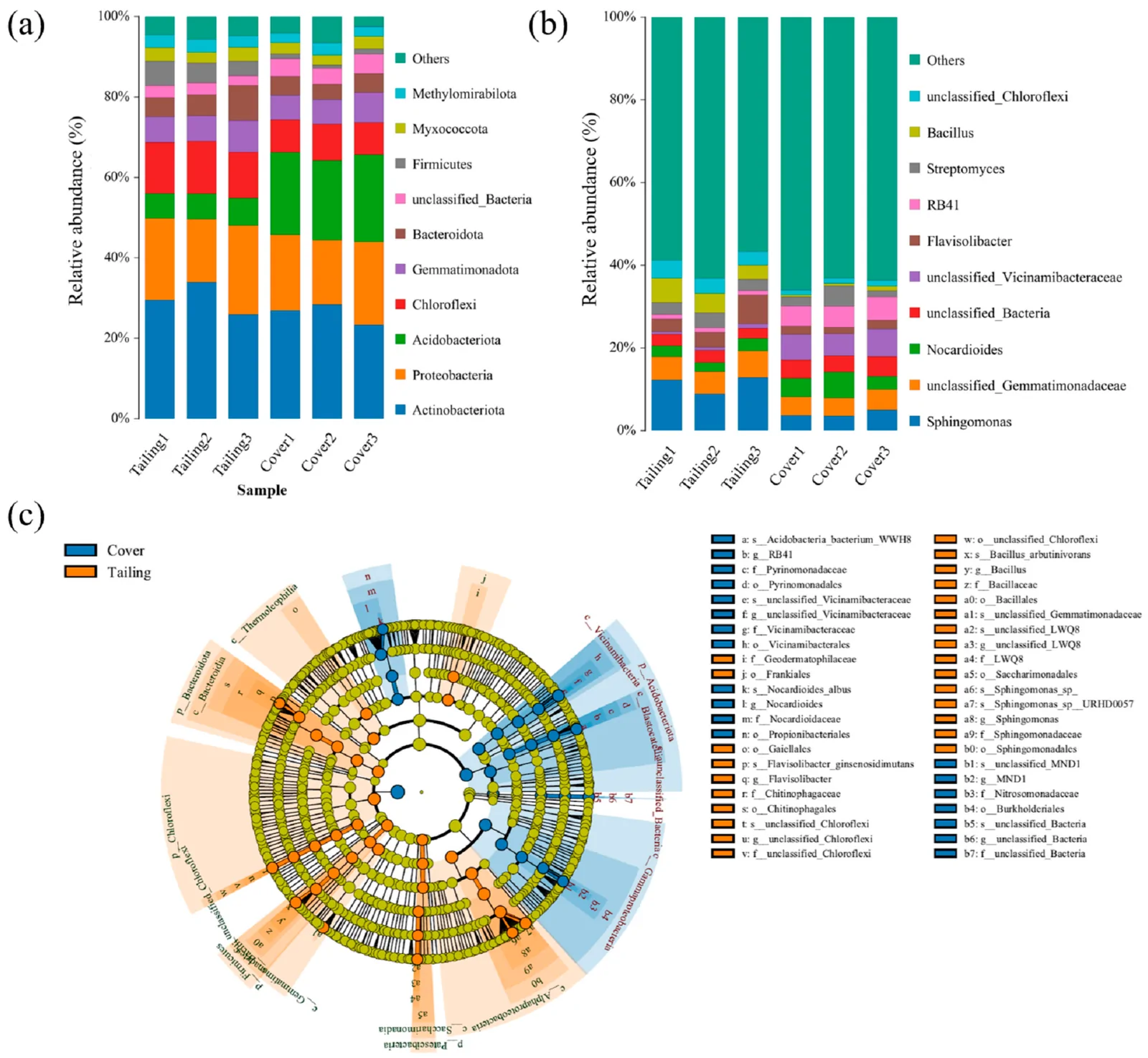
Relative abundance of bacterial communities at (**a**) the phylum level and (**b**) the genus level; (**c**) LEfSe cladogram identifying significant microbial biomarkers (linear discriminant analysis score > 2.0) that discriminate between tailings-contaminated soil (orange) and cover soil (blue) communities across different taxonomic levels.

At the phylum level (Figure 8a), the bacterial communities in both soil types were dominated by Actinobacteriota, Proteobacteria, Acidobacteriota, and Chloroflexi. However, Acidobacteriota was more prevalent in the cover soil. At the genus level (Figure 8b), specific taxa showed distinct distribution patterns. *Sphingomonas* (Proteobacteria) was markedly more abundant in the tailings-contaminated soil. Conversely, genera such as *Nocardioides* (Actinobacteria), RB41 (Phylum Acidobacteriota, Class Blastocatellia), and members of the family Vicinamibacteraceae (Phylum Acidobacteriota) were more dominant in the cover soil. LEfSe cladogram analysis (Figure 8c) demonstrated a significant shift in rhizosphere microbial composition between the cover soil and the tailings-contaminated soil. The cover soil contained a higher abundance of biomarkers within Acidobacteriota (e.g., Vicinamibacteraceae and Pyrinomonadaceae), Gammaproteobacteria (e.g., Burkholderiales, Nitrosomonadaceae, and *MND1*), and specific Actinomycetota lineages (e.g., Nocardioidaceae and *Nocardioides*). Conversely, the tailings-contaminated soil was enriched in biomarkers, notably within Bacteroidota (e.g., Chitinophagaceae and *Flavisolibacter*), Bacillota (e.g., Bacillaceae and *Bacillus*), Chloroflexi, Alphaproteobacteria (e.g., Sphingomonadaceae and *Sphingomonas*), as well as Actinomycetota orders such as Frankiales and Gaiellales.

**Figure 9:**
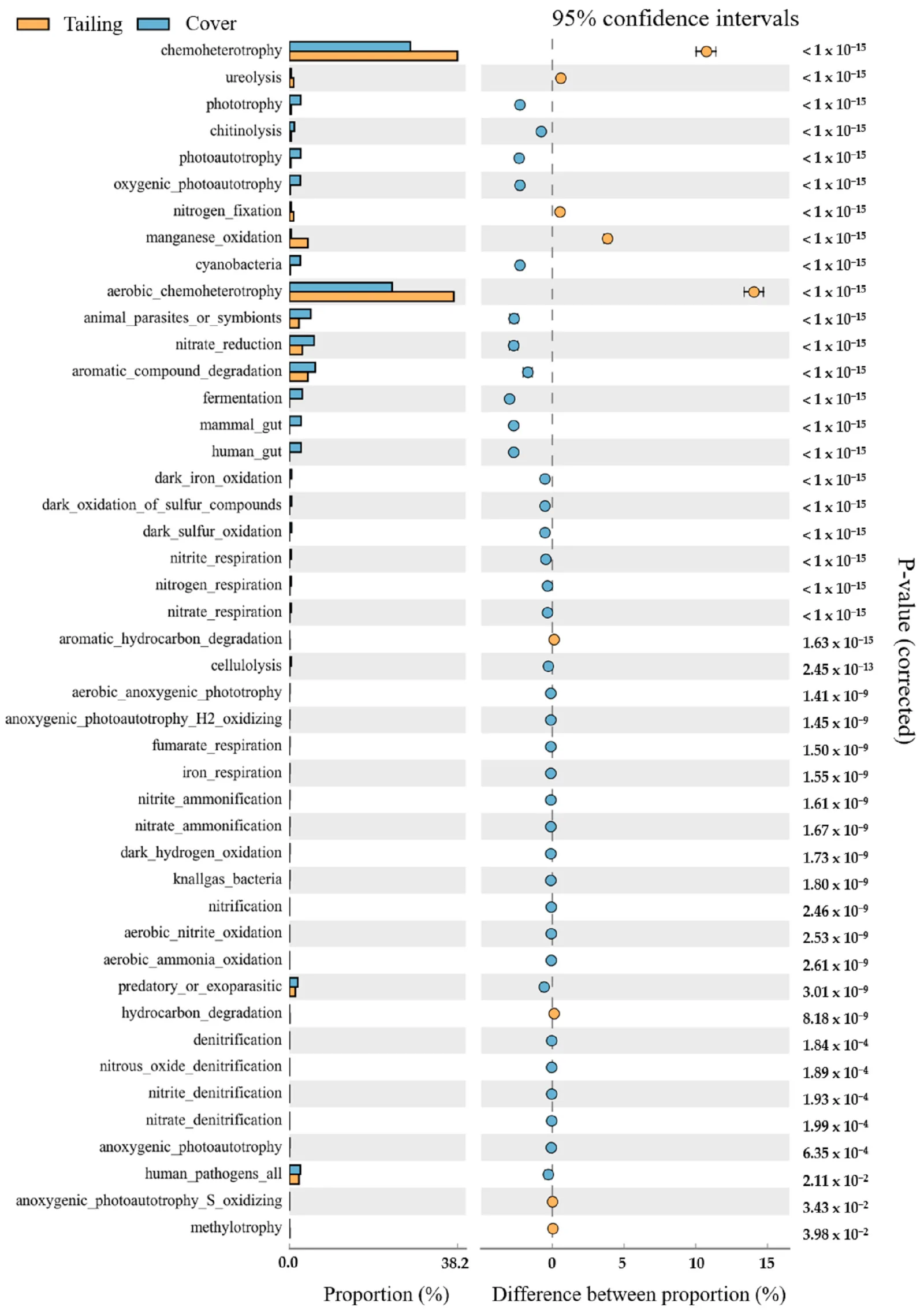
FAPROTAX functional prediction of bacterial communities in tailings-contaminated soil (orange) and cover soil (blue). The bar plot (**left**) shows the relative proportion of each functional group, while the dot plot (**right**) shows differences between proportions with 95% confidence intervals and corrected *p*-values.

Functional annotation using the FAPROTAX database suggested potential shifts in the predicted metabolic potential of the bacterial communities between the tailings-contaminated soil and the cover soil (Figure 9). The rhizosphere communities were dominated by chemoheterotrophy and aerobic chemoheterotrophy, with the tailings-contaminated soil showing a higher proportion of these functions. In addition, the tailings-contaminated soil exhibited a distinct functional signature characterized by significantly higher levels of ureolysis, nitrogen fixation, and manganese oxidation. In contrast, the cover soil showed markedly higher predicted proportions of phototrophy and photoautotrophy, alongside a greater prevalence of nitrate reduction and fermentation. The cover soil also showed significantly higher levels of chitinolysis and cellulolysis.

**Figure 10:**
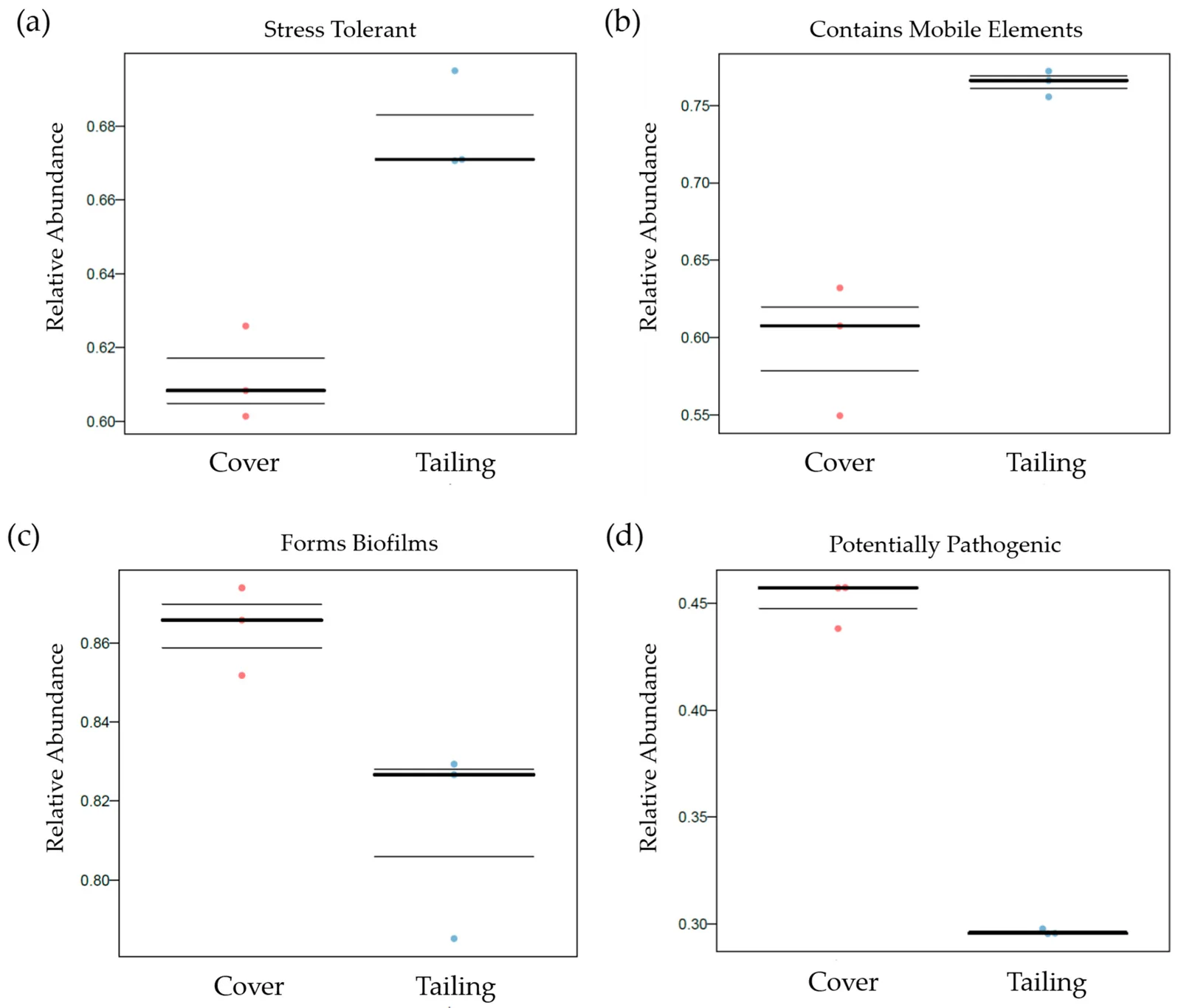
Predicted phenotypic traits of microbial communities using BugBase: (**a**) stress tolerance, (**b**) abundance of mobile genetic elements, (**c**) biofilm formation potential, and (**d**) potential pathogenicity. Differences in predicted relative abundance may reflect adaptive strategies of bacteria in tailings-contaminated soil versus cover soil.

BugBase analysis was employed to predict the functional phenotypes of the bacterial communities in the tailings-contaminated soil and the cover soil, and the results suggested potential differences in phenotypic composition between the two environments (Figure 10). The bacterial community in the tailings-contaminated soil showed higher predicted relative abundances of stress-tolerant phenotypes (Figure 10a) and taxa hypothesized to contain mobile genetic elements (Figure 10b) compared to the cover soil. In contrast, the cover soil community showed higher predicted abundances of biofilm formation (Figure 10c) and potential pathogenicity (Figure 10d). Overall, these predictions suggest possible adaptive traits in the rhizosphere bacterial communities.

## 3. Discussion

Plants in the genus *Cyanotis* exhibit morphological plasticity in response to environmental conditions, allowing them to thrive in habitats ranging from moist to arid areas [13,15,29]. Therefore, species identification within this genus should use a combination of morphological and genetic data. The *rbcL* gene is readily amplified and aligned across a wide range of land plants, whereas the non-coding *trnH–psbA* spacer providing sufficient sequence variation to differentiate closely related species when morphological features are unclear [30,31]. In this study, the plant sample was identified as *Cyanotis arachnoidea* through morphological characterization (Figure 1) and DNA barcoding analysis using the chloroplast markers *rbcLa* and *trnH-psbA* (Figure 2). Furthermore, the sequence alignment results obtained via NCBI BLASTN search support the utility of DNA barcoding for plant species identification [32].

Rehabilitation of the Phadaeng zinc mine requires addressing both the old tailings pile and the open-pit mine area, the latter of which must be covered with non-contaminated soil prior to restoration. In this study, tailings-contaminated soil had higher C, N, and mineral (Fe, P, and K) contents than cover soil (Table 1), possibly because the soil originated from a tailings pile that had been covered by vegetation for a long period [4,33]. Total Cd in the cover soil (101.74 mg kg^−1^) did not exceed Thailand’s soil quality standards for agricultural and living purposes (Type 2), which specify a maximum limit of 762 mg kg^−1^. Conversely, total Cd in the tailings-contaminated soil reached 865.07 mg kg^−1^, exceeding this regulatory threshold and highlighting the persistence of high Cd contamination at the former mine tailings site. However, Thailand’s soil quality standard B.E. 2564 (2021) does not regulate the standard concentration for Zn [34]. Concentrations of these metals and minerals (P, K, and Fe) were lower at Day 60 than at the initial baseline (Day 0) (Table 1), which may partly reflect Zn and Cd accumulation by *C. arachnoidea* (Figure 5) and plant growth (Figure 4) [14,35]. In contrast, organic matter content increased in all soil types (untreated and autoclaved cover and tailings soils) at Day 60 (Table 1) [8]. In addition, the decrease in soil pH and the increase in extractable Zn content and C:N ratio (Table 1) might have resulted from the combined activity of plants and soil microorganisms [36,37,38].

*C. arachnoidea* growing in tailings-contaminated soil showed symptoms of metal stress, including reddened leaves, larger stomata and decreased stomatal density, reduced leaf number, lack of branch development, and underdeveloped roots (Figure 3 and 4a–c). High levels of zinc interfere with cell division or elongation, resulting in smaller and/or fewer leaves [5]. Cd inhibits cell division and elongation in both apical and axillary meristematic tissues [7]. Excessive Zn and Cd accumulated in plant cells can affect the roots, causing black, decayed root tips, reduced length, and altered root hair morphology [5,7]. Plant stomata are affected by Zn and Cd toxicity, with the specific response depending on the plant species [39]. Larger stomata were observed in *Murdannia spectabilis* (Kurz) Faden growing in Zn- and Cd-contaminated soil [40]. In *C. arachnoidea* grown in tailings-contaminated soil, high shoot accumulation of Zn and Cd (Figure 5a,b) may have altered epidermal development, resulting in a reduction in stomatal density alongside a compensatory increase in stomatal size (Figure 4b) [41,42]. Although an increased stomatal size may serve as a structural trade-off to maintain baseline gas exchange, its functional efficacy regarding CO_2_ assimilation and water balance may be severely compromised under heavy metal stress [43,44]. In addition, larger stomata under metal toxicity often exhibit sluggish movement kinetics and impaired guard cell regulation due to reactive oxygen species (ROS)-mediated damage and disrupted ion channel activity [45,46]. The reduction in leaf size and plant growth (Day 60) (Figure 3), the trend toward lower mean fresh weights (Figure 4c), and the decrease in total chlorophyll content (Figure 4d) are consistent with this interpretation.

The red leaf coloration might be associated with increased anthocyanin content (Figure 4e). Plants produce anthocyanins under both Zn and Cd stress [47]. High Zn levels can induce oxidative stress and leaf discoloration, shifting from red to purple due to increased anthocyanin levels, as observed in *Coriandrum sativum* L. [48] and *Zea mays* L. [49]. In addition, reddening of *Raphanus sativus* L. leaves was associated with the role of the *RsDof* gene in the response to Cd stress and anthocyanin biosynthesis [50]. Zn and Cd stress typically triggers the production and accumulation of proline and antioxidant enzymes, such as SOD (superoxide dismutase), CAT (catalase), and POD (peroxidase) [51,52,53,54,55]. In this study, however, *C. arachnoidea* growing in tailings-contaminated soil produced significantly lower proline levels (Figure 4e), whereas TPC and TFC exhibited significant variations across soil treatments (Figure 4f). Exposure to Zn and Cd can induce gene expression within the anthocyanin biosynthesis pathway, helping to sequester excess metal ions and reduce the production of ROS [56,57]. Anthocyanins can bind with Zn and Cd to form anthocyanin–metal complexes, which are then sequestered in the vacuoles of epidermal cells to isolate toxic metals from photosynthetic tissues [49,58]. Furthermore, since both anthocyanins and proline can act as intracellular osmoregulators, the marked accumulation of anthocyanins observed in this study may have compensated for the reduced proline levels to maintain cellular water balance [56]. Plants can utilize vacuolar anthocyanins as a primary non-enzymatic defense mechanism to scavenge ROS during the initial stages of stress; if anthocyanin levels are insufficient, plants trigger the synthesis of proline and antioxidant enzymes to work synergistically [56,59,60], depending on metal concentration and the duration of exposure [54]. These findings suggest that, in *C. arachnoidea,* anthocyanin induction may serve as a major defense pathway against severe metal toxicity.

Excess Zn can replace Mg^2+^ in chlorophyll molecules, reducing photosynthetic pigment accumulation [5], whereas Cd directly suppresses chlorophyll biosynthesis [7]. In agreement with these mechanisms, total chlorophyll content in *C. arachnoidea* was significantly lower in plants grown in both untreated and autoclaved tailings-contaminated soil compared to those in untreated cover soil (Figure 4d). However, despite the reduction in total chlorophyll, chlorophyll *a* content remained relatively stable across treatments, whereas chlorophyll *b* content declined significantly, particularly under autoclaving—leading to a marked increase in the chlorophyll *a*/*b* ratio (Figure 4d). The relative stability of chlorophyll *a* and overall photosynthetic maintenance under metal stress may be attributed to stimulated anthocyanin production under metal stress conditions (Figure 4e). Anthocyanins can help prevent chlorophyll degradation, scavenges ROS, and chelates metals, thereby protecting chloroplasts and photosystem II (PSII) from damage [49,56,58]. Interestingly, autoclaving resulted in a significant decrease in chlorophyll *b* content in both soil types (Figure 4d). This may be attributed to autoclaving, which significantly reduced the initial bacterial population as well as the diversity (*H*) and richness (*S*) of rhizosphere bacteria (Table 2). The loss or suppression of these beneficial rhizosphere microorganisms, particularly potential plant growth-promoting bacteria (PGPB), likely compromised their functional role in promoting photosynthetic pigment accumulation and chlorophyll retention under heavy-metal stress conditions [61,62,63].

Based on the uptake and translocation profiles of Zn and Cd in *C. arachnoidea* grown in the untreated and autoclaved cover and tailings-contaminated soils for 60 days (Figure 5), the parameters for metal accumulation and translocation can be summarized as follows. For Zn, *C. arachnoidea* exhibited a phytostabilization profile (TF < 1, BAF_root_ > 1, and BAF_shoot_ > 1), with shoot and root Zn concentrations reaching 3.07 g kg^−1^ DW and 14.02 g kg^−1^ DW, respectively. For Cd, the species displayed a high-translocating profile (TF > 1, BAF_root_ < 1, and BAF_shoot_ < 1), with shoot and root Cd concentrations of 0.31 mg kg^−1^ DW and 0.03 mg kg^−1^ DW, respectively. Plant metal hyperaccumulation and suitability for phytoextraction can be evaluated based on the established threshold criteria (Zn ≥ 10,000 mg kg^−1^ DW in shoot, TF > 1, and BAF_shoot_ > 1) [64,65]. Conversely, species exhibiting TF < 1 alongside BAF_root_ > 1 are characterized as potential candidates for phytostabilization [65]. To evaluate plant potential for Cd phytoextraction, three standard threshold criteria must be met simultaneously (Cd ≥ 100 mg kg^−1^ DW in shoot, TF > 1, and BAF_shoot_ > 1) [64,66]. Species exhibiting TF > 1 alongside BAF_shoot_ < 1 or low tissue concentrations are generally categorized as high-translocating excluders rather than true Cd hyperaccumulators [65,67]. Based on these criteria, alongside the assessment of growth characteristics and comparative physiology between plants grown in cover and tailings-contaminated soils (Figure 4 and Figure 5) [68], *C. arachnoidea* can be classified as a highly Zn-tolerant phytostabilizer and a high-translocating Cd excluder. However, these distinct profiles may be partly attributed to competitive transport dynamics and specific transporter preferences between Zn and Cd. Therefore, the inherent phytoremediation potential for Zn and/or Cd in *C. arachnoidea* should be further verified under controlled conditions, using single-element (Zn or Cd) and combined-element (Zn + Cd) exposures.

Plant survival in soils with high metal concentrations and metal accumulation in plant biomass are influenced by soil microorganisms [22], particularly metal-tolerant rhizosphere strains [21,28]. Accordingly, the lower accumulation of Zn and Cd in *C. arachnoidea* grown in autoclaved soil (Figure 5), along with the significant decrease in chlorophyll *b*, altered anthocyanin levels, the significant decline in proline content, and the marked reduction in TPC alongside significant variations in TFC (Figure 4d–f), may be attributed to partial microbial suppression (Table 2). Therefore, rhizosphere soil samples from plants grown in the untreated cover and tailings-contaminated soils were analyzed for bacterial community structure. The culture-dependent findings (Table 2) are broadly consistent with the HTS data. Alpha diversity indices (Shannon and Chao1) revealed that the rhizosphere soil of plants grown in tailings-contaminated soil had lower bacterial richness and evenness (Figure 7b,c), which was consistent with previous studies showing that heavy metal stress suppresses overall microbial diversity [69,70]. Beta diversity analysis revealed a clear separation between the two groups (Figure 7e,f), with metal contamination likely being the primary cause [69,71]. Furthermore, the higher phylogenetic diversity observed in the tailings-contaminated soil (Figure 7d) indicated that the metal-contaminated rhizosphere harbored bacteria from evolutionarily distinct lineages [71], possibly reflecting diverse adaptive strategies to cope with extreme metal stress [38,72].

Isolate identification via 16S rRNA gene phylogeny (Figure 6) demonstrated that the majority of strains isolated from the cover soil (CSCT isolates) belonged to Firmicutes (e.g., *Fictibacillus* and *Staphylococcus*) and Proteobacteria (e.g., *Pseudomonas* and *Rhizobium*), which commonly predominate in unpolluted or control soils [73]. In contrast, the majority of strains isolated from the tailings-contaminated soil (TSCT isolates) belonged to Actinomycetota (e.g., *Arthrobacter, Pseudarthrobacter*, *Nocardia*, and *Nocardioides*) and Bacteroidota (e.g., *Pontibacter*). The enrichment of Actinomycetota and Bacteroidota in heavy metal-contaminated environments is well-documented [70,74]. Analysis of the relative abundance at the phylum level (Figure 8a) confirmed the presence of taxa identified via culture-based methods and further revealed that Chloroflexi were more prevalent in tailings-contaminated soil, whereas Acidobacteriota were more prevalent in cover soil. These phyla have been reported in mining areas with high levels of metal contamination in the soil [70,72,75,76]. Furthermore, the rhizosphere soils harbored a diverse set of bacteria that have not been assigned to any specific genus (Figure 8b). This large proportion of unclassified taxa reflects the large, still poorly characterized microbial diversity and the highly complex nature of the rhizosphere ecosystem, wherein many ASVs cannot yet be distinguished at the genus level using current databases [25].

The LEfSe cladogram analysis demonstrated a distinct shift in rhizosphere bacterial composition, consistent with selection by heavy metals (Figure 8c). In tailings-contaminated soil, significant enrichment of Bacillota (*Bacillus*), Alphaproteobacteria (*Sphingomonas*), Bacteroidota, Chloroflexi, and Actinomycetota (*Frankiales* and *Gaiellales*) may reflect an adaptive response to severe Zn/Cd stress. Genera such as *Bacillus* and *Sphingomonas* are well known for metal tolerance and for producing extracellular polymeric substances (EPS) that immobilize toxic metals [21,28], while Bacteroidota and Chloroflexi thrive under oligotrophic, high-stress conditions [24]. Conversely, cover soil was enriched with taxa involved in nutrient cycling and plant–microbe interactions. The prevalence of Acidobacteriota (Vicinamibacteraceae and Pyrinomonadaceae) reflects a stable, organic-rich environment [69,77]. Furthermore, key nitrogen-cycling biomarkers—including ammonia-oxidizing Gammaproteobacteria (Nitrosomonadaceae and MND1) and Burkholderiales [70,78]—alongside carbon-decomposing Actinomycetota (Nocardioides) [79,80] suggest a functionally diverse rhizosphere that supports nutrient turnover and plant growth.

FAPROTAX functional profiling (Figure 9) indicated predicted enrichments of chemoheterotrophic and aerobic functions across both rhizosphere soil types (cover and tailings-contaminated soils), consistent with typical root-associated microbiomes [73]. A predicted shift toward survival-oriented functions (nitrogen fixation, ureolysis, and manganese oxidation) in the tailings-contaminated soil suggested pathways that might respond to heavy metal toxicity and nutrient deprivation [22,78,81]. Conversely, higher predicted ecosystem-supporting functions (phototrophy, cellulolysis, and nitrate reduction) in the cover soil may reflect a functional profile geared toward organic matter decomposition and biogeochemical cycling [6,70,82]. Ultimately, this functional divergence suggests that heavy metal contamination may shape the functional potential and metabolic versatility of the *C. arachnoidea* rhizosphere. BugBase phenotypic predictions suggested potential variations in functional profiles between the two soil environments (Figure 10). Rhizosphere communities in tailings-contaminated soil showed a predicted enrichment of stress-tolerant phenotypes and taxa containing mobile genetic elements. These predicted traits might reflect metal-driven selection [70] and could suggest a possible role for horizontal gene transfer (HGT) in disseminating heavy metal resistance genes [28,72]. Conversely, cover soil exhibited higher predicted abundances of biofilm and potentially pathogenic phenotypes, which may reflect root–microbe colonization supported by root exudates [23,83]. In contrast, heavy metal toxicity in tailings-contaminated soil could have constrained these predicted opportunistic and biofilm-forming groups [24,70,71]. Because 16S-based functional inferences remain strictly hypothetical, future metatranscriptomic and multi-omic studies would be required to confirm whether these predicted pathways represent actual in situ adaptive strategies.

## 4. Materials and Methods

### 4.1. Plant Collection and Molecular Identification

*Cyanotis* specimens were collected from Phu Kradueng National Park, Loei Province, Thailand (16°53′47.83″ N, 101°47′35.77″ E), and a voucher specimen (MSUT-8768) was deposited at Mahasarakham University Herbarium. Leaf tissue (100 mg) was ground in liquid nitrogen and extracted using a modified cetyltrimethylammonium bromide (CTAB) protocol [84]. Two chloroplast DNA barcode loci, *rbcLa* and *trnH–psbA*, were amplified using primer pairs commonly employed in plant DNA barcoding studies. The *rbcLa* locus was amplified using *rbcLa* forward 5′–ATGTCACCACAAACAGAGACTAAAGC–3′ and *rbcLa* reverse 5′–GTAAAATCAAGTCCACCRCG–3′, whereas the *trnH–psbA* region was amplified using *trnH*f05 forward 5′–CGCGCATGGTGGATTCACAATCC–3′ and *psbA*3f reverse 5′–GTTATGCATGAACGTAATGCTC–3′ primers [85]. PCR was performed using 2× ViRed Taq Master Mix (Vivantis technoligies Sdn. Bhd., Subang Jaya, Malaysia). PCR amplicons were purified using the MEGAquick-spin^TM^ plus Kit (iNtRON Biotechnology Inc., Seongnam-Si, Republic of Korea) and sequenced Macrogen, Inc. (Seoul, Republic of Korea). Sequences were trimmed and assembled using Pregap4 and Gap4 in the Staden Package [86]. Species identification was verified via NCBI BLAST v2.17.0 [87]. Phylogenetic analyses were conducted in MEGA version 12 [88] using Maximum Likelihood (ML; Tamura 3-parameter model with Invariable Sites, T92+I) and Neighbor-Joining (NJ; Kimura 2-parameter model) methods, each with 1000 bootstrap replicates. The nucleotide sequences were deposited in NCBI GenBank under accession numbers PX828449 for Cyano-PKD-rbcLa and PX991845 for Cyano-PKD-trnH-psbA.

### 4.2. Plant Material and Cultivation

The collected *Cyanotis* plants were grown at Sakon Nakhon Rajabhat University (17°11′10.5″ N, 104°05′15.8″ E) in a naturally ventilated passive greenhouse with plastic-film side walls and mesh-covered A-frame roof openings, operating without artificial climate control. For tissue culture propagation, young stems were washed and surface-sterilized with 75% (*v*/*v*) ethanol for 3 min, followed by 0.9% (*v*/*v*) sodium hypochlorite (NaOCl) for 10 min and 0.3% (*v*/*v*) NaOCl for 5 min, then rinsed three times with sterile distilled water for 5 min each. The surface-sterilized explants were trimmed and transferred to Murashige and Skoog (MS) medium [89], supplemented with 0.1 mg L^−1^ 6-benzylaminopurine (BAP). Cultures were maintained at 25 °C with a 16-h photoperiod at 1800 lux. After 30 days, the mother plants were separated and subcultured onto the MS medium to obtain uniform plantlets for the pot experiment.

### 4.3. Soil Sample and Soil Analysis

Soil samples were collected from the former Phadaeng zinc mine in Phra That Pha Daeng Sub-District, Mae Sot District, Tak Province. Tailings-contaminated soil was collected from a former tailings disposal site (16°39′47.6″ N, 98°38′41.9″ E), and cover soil was collected from the topsoil layer imported to backfill and cap the former open-pit mining area for post-mining ecological restoration (16°39′25.6″ N, 98°39′44.7″ E). For each soil type, a composite sampling strategy was employed by collecting topsoil (0–15 cm depth). Six subsamples were collected across the study plot using a systematic 6-point grid pattern. The collected soil samples were thoroughly pooled, homogenized, air-dried in the shade, crushed, and sieved through a 2 mm plastic sieve (10 U.S. mesh size). The treatments designated as ‘Autoclaved cover soil’ and ‘Autoclaved tailings-contaminated soil’ were prepared by subjecting the soils to three 30-min autoclaving cycles (121 °C) at 6-h intervals. This triple-autoclaving process was applied to achieve partial soil sterilization (microbial reduction), as soil matrices naturally shelter heat-resistant, spore-forming microorganisms. This treatment was intended to significantly suppress and disturb the native soil microbial biomass to assess plant growth under reduced-microbiome conditions. Since sterilization was not the primary objective, testing to reach a state of no colony growth was not performed. Total Zn, Cd, and Fe were quantified using flame atomic absorption spectrometry (FAAS) (240FS AA, Agilent Technologies Australia Pty Ltd., Mulgrave, Australia) after aqua regia digestion [90]. DTPA-extractable Zn and Cd were determined using 0.005 M diethylenetriaminepentaacetic acid (DTPA) [91]. Soil pH was measured in a 1:1 (*w*/*v*) soil-to-water suspension [92]. Total nitrogen (N) was determined by automated Kjeldahl digestion and titration (FOSS Kjeltec™ 8200, FOSS, Hilleroed, Denmark). Organic carbon (OC) was quantified using the Walkley-Black wet oxidation method [93]. Soil organic matter (OM) and the C:N ratio were calculated based on the obtained OC and total N concentrations. Available phosphorus (P) was extracted using the Bray II method and measured spectrophotometrically at 882 nm [94]. Available potassium (K) was extracted with 1 M ammonium acetate (NH_4_CH_3_CO_2_, pH 7.0) and analyzed using FAAS [92]. All chemicals used were of analytical grade.

### 4.4. Pot Experiment

One-month-old tissue culture-derived plantlets were gently rinsed under running tap water and then immersed in 0.4% (*v*/*v*) Physan 20 (Maril Products, Inc., Tustin, CA, USA) for 1 h to prevent root rot after transplantation. The cleaned plantlets were transferred to commercial peat moss (Chia-Tai Co., Ltd., Bangkok, Thailand) mixed with perlite (30:1 ratio) and placed in a growth chamber for 5 days to acclimate, before being further grown in a nursery for another 25 days. Subsequently, plants of uniform size were selected for the pot experiment. Roots were gently washed under running tap water to remove residual peat moss prior to cultivation in four distinct soil treatments: cover soil, autoclaved cover soil, tailings-contaminated soil, and autoclaved tailings-contaminated soil (hereafter referred to as ‘tailing soil’ and ‘autoclaved tailing soil’, respectively, in tables and figures). Each plant was grown in a plastic pot (12 cm in diameter and 10 cm deep) containing 460 g of the respective soil type, with 10 replicates per treatment. The pot experiment was conducted for 60 days (August–October 2024) in the naturally ventilated, passive greenhouse. Plants were irrigated every two days with 100 mL of sterilized water per pot, and the soil surface was lightly scarified every 10 days to enhance soil aeration. At the end of the experiment, soil from each replicate pot was thoroughly mixed and composite samples were prepared using the coning and quartering method. Briefly, the soil was formed into a symmetrical conical pile, divided into four equal quarters, and two diagonally opposite quarters were combined to yield a 150 g composite soil sample, which was stored in a polyethylene Ziplock bag at 4 °C prior to analysis.

Soil temperature and moisture content were monitored using a wireless soil meter (Tuya Zigbee IP67, Shenzhen Yieryi Technology Co., Ltd., Shenzhen, China), while canopy-level light intensity and temperature were measured using a digital thermo-lux meter (Wintact WT81, Shenzhen Wintact Electronics Co., Ltd., Shenzhen, China). During the 60-day experimental period, greenhouse temperature and light intensity fluctuated within ranges of 25–32 °C and 12,000–60,000 lux, respectively. Soil temperature fluctuated between 24 and 35 °C, while moisture content ranged from 36% to 76%. Local weather data for the experimental period were obtained from the Sakon Nakhon Meteorological Station; temperatures ranged from 24 °C to 33 °C (average 28 °C), and relative humidity ranged from 64% to 94% (average 87%).

### 4.5. Plant Analysis

Plants grown in the four different soil types were evaluated for their morphological characteristics, Zn and Cd contents in the biomass, phytochemical responses, and rhizosphere bacterial diversity.

#### 4.5.1. Plant Morphology and Leaf Micromorphology

Digital images of the entire plants were acquired on days 0, 30, and 60 to monitor growth progress. Following the 60-day growth period, plants were harvested and evaluated for leaf number, branch development, leaf coloration, and root morphological traits. To study leaf epidermal anatomy, the foliar epidermis of adaxial (upper) and abaxial (lower) surfaces of fresh, healthy mature leaves was prepared using the impression technique [95]. A small portion of Quickfix (Wembley Enterprises Ltd., New Delhi, India) was applied to the leaf surface and allowed to dry for 15 min. The leaf epidermis was gently peeled and placed on a glass slide. Stomatal density, stomatal size (length × width), and trichome characteristics were recorded using a light microscope (Leica DM750, Leica Microsystems GmbH, Wetzlar, Germany) coupled with Leica Enersight Desktop 1.5.1 Microscope Software.

#### 4.5.2. Plant Biomass and Zn/Cd Accumulation

The plants were gently removed from the pots, cleaned with a mild detergent (Lipon F, Lion Corporation (Thailand) Ltd., Si Racha, Thailand), thoroughly rinsed under running tap water, and finally rinsed with deionized (DI) water. Clean plants were blotted dry before fresh weight was measured. Dry weight was determined after drying the samples at 60 °C for 24 h in a hot-air oven (Universal Oven UF30, Memmert GmbH + Co. KG, Büchenbach, Germany). Plant samples were separated into root and shoot parts prior to freeze-drying (LaboGene A/S, Allerød, Denmark). Accumulation of Zn and Cd in plant tissues was determined by digesting the freeze-dried samples using a modified nitric-perchloric acid wet digestion method [96]. Briefly, 0.1 g of plant sample was digested in 3 mL of 70% (*v*/*v*) HNO_3_ at 150 °C for 1 h; subsequently, 1 mL of 70% (*v*/*v*) HClO_4_ was added and heated at 215 °C for 2 h. After cooling, 3 mL of DI water was added, and the solution was heated at 90 °C for 1 h. The digested solutions were analyzed using FAAS. Metal concentrations in shoots and roots were expressed on a plant dry weight (DW) basis. Total metal accumulation per plant (mg plant^−1^), translocation factor (TF), and bioaccumulation factor (BAF) were calculated using Equations (1), (2), and (3), respectively [3]:

*Total metal accumulation per plant* = (*C_shoot_* × *DW_shoot_*) + (*C_root_* × *DW_root_*) (1)

(2) $$\mathrm{Translocation}\;\mathrm{factor}\;\left(\mathrm{TF}\right)=\frac{\mathrm{Concentration}\;\mathrm{of}\;\mathrm{metal}\;\mathrm{in}\;\mathrm{shoots}\;(\mathrm{mg}\;kg^{-1}\mathrm{DW})\;}{\mathrm{Concentration}\;\mathrm{of}\;\mathrm{metal}\;\mathrm{in}\;\mathrm{roots}\;(\mathrm{mg}\;kg^{-1}\mathrm{DW})\;}$$

(3) $$\mathrm{Bioaccumulation}\;\mathrm{factor}\;\left(\mathrm{BAF}\right)=\frac{\mathrm{Concentration}\;\mathrm{of}\;\mathrm{metal}\;\mathrm{in}\;\mathrm{biomass}\;(\mathrm{mg}\;kg^{-1\;}\mathrm{DW})}{\mathrm{Available}\;\mathrm{concentration}\;\mathrm{of}\;\mathrm{metal}\;\mathrm{in}\;\mathrm{soil}\;(\mathrm{mg}\;kg^{-1}\;\mathrm{DW})}$$

where *C_shoot_* and *C_root_* represent the metal concentrations (mg g^−1^ DW) in the shoot and root tissues, and *DW_shoot_* and *DW_root_* denote the respective dry weight per plant (g DW plant^−1^) of the shoot and root.

#### 4.5.3. Phytochemical Content and Antioxidant Activities

Chlorophyll content: Chlorophylls were extracted from fresh leaves (0.1 g) using 5 mL of 95.5% (*v*/*v*) acetone at 4 °C for 48 h in the dark [97]. Absorbance was measured at 662 and 644 nm, and concentrations of chlorophyll *a* (Chl *a*), chlorophyll *b* (Chl *b*) and total chlorophyll (Chl *a + b*) were calculated and expressed as mg g^−1^ fresh weight (FW).

Proline content: Proline was quantified following the method described in [98], with modifications. Fresh leaves were homogenized in 3% (*w*/*v*) sulfosalicylic acid (C_7_H_6_O_6_S) and centrifuged at 6000 rpm for 20 min. The supernatant (1 mL) was reacted with 0.5 mL of acid-ninhydrin reagent and 0.5 mL of glacial acetic acid at 100 °C for 1 h, then placed on ice for 15 min to stop the reaction. After extraction with 4 mL of toluene, the absorbance of the organic phase was recorded at 520 nm. Proline content was expressed as µg g^−1^ FW using a proline standard curve.

Anthocyanin content: Monomeric anthocyanins were extracted from fresh leaves (1 g) using 4 mL of 0.1 N HCl in 96% (*v*/*v*) ethanol (1:9 ratio) for 1 h and quantified via the pH-differential method at pH 1.0 (0.025 M KCl) and pH 4.5 (0.4 M CH_3_COONa) [99]. Absorbance was measured at 520 and 700 nm. Total monomeric anthocyanin content was calculated and reported as µg g^−1^ FW.

Total phenolic content (TPC): Freeze-dried leaves were extracted with 70% (*v*/*v*) ethanol under ultrasound-assisted extraction at 50 °C for 1 h. TPC was determined using a modified Folin-Ciocalteu micro-method [100]. Briefly, 100 µL of the sample was mixed with 500 µL of 10% (*w*/*v*) Folin-Ciocalteu reagent, 100 µL of 7.5% (*w*/*v*) Na_2_CO_3_, and 300 µL of DI water. The mixture was incubated in the dark for 2 h, and the absorbance was measured at 731 nm. TPC was expressed as mg gallic acid equivalents per gram dry weight (mg GAE g^−1^ DW).

Total flavonoid content (TFC): TFC was analyzed using a modified aluminum trichloride colorimetric method [101]. Briefly, 100 µL of the sample was mixed with 500 µL of DI water and 30 µL of 5% (*w*/*v*) NaNO_2_, followed by incubation in the dark for 5 min. Subsequently, 60 µL of 10% (*w*/*v*) AlCl_3_ was added, and the mixture was further incubated for 6 min, before being neutralized with 200 µL of 1 M NaOH and 110 µL of DI water. Absorbance was measured at 510 nm after a final 5-min incubation. TFC was expressed as mg epicatechin equivalents per gram dry weight (mg EPE g^−1^ DW).

Free radical scavenging activity (FRSA): Antioxidant activity was evaluated using the 2,2-diphenyl-1-picrylhydrazyl (DPPH) radical scavenging assay [102]. Briefly, 100 µL of the sample was mixed with 900 µL of 0.08 mM DPPH solution. The mixture was incubated in the dark for 30 min, and the absorbance was measured at 515 nm to calculate the DPPH radical scavenging percentage (%).

### 4.6. Rhizosphere Bacterial Community and Diversity

Rhizosphere soil tightly adhering to plant roots was collected following the method described in [103]. Soil samples were divided into two portions: one stored at 4 °C for culture-dependent bacterial analyses and the other stored at −80 °C for high-throughput sequencing.

#### 4.6.1. Culturable Bacterial Diversity and Identification

A soil sample (5 g) was suspended in 95 mL of 0.9% (*w*/*v*) NaCl solution, shaken at 180 rpm for 10 min, and serially diluted prior to plating on Tryptic Soy Agar (TSA) supplemented with 100 mg L^−1^ nystatin. Plates were incubated at 30 °C for 72 h, and bacterial abundance was expressed as log CFU g^−1^ dry soil. Colony morphology was characterized according to standard microbiological criteria [104]. Alpha diversity indices, including the Shannon-Wiener index (*H*), richness (*S*), and evenness (*E_H_*), were calculated [105]. Representative isolates were identified by partial 16S rRNA gene sequencing using universal primers 27F (5′–AGAGTTTGATCMTGGCTCAG–3′) and 1492R (5′–GGTTACCTTGTTACGACTT–3′) (Macrogen, Republic of Korea). PCR products were purified, sequenced, and compared against the NCBI database using BLASTN. Species-level identification was defined at >98.8% 16S rRNA sequence similarity with distinct genetic distances to reference strains [106]. Maximum Likelihood (ML) and Neighbor-Joining (NJ) phylogenetic trees were constructed using MEGA version 12 [88] under the Kimura 2-Parameter + Gamma + Invariant Sites (K2+G+I, selected by BIC) and Kimura 2-Parameter substitution models, respectively, with 1000 bootstrap replicates. Sequence data were deposited in the NCBI GenBank database under accession numbers PX956716, PX956800-PX956801, PX956829, PX956838, PX956946-PX956947, PX956987-PX956988, PX956990, PX957011-PX957013, and PX957282.

#### 4.6.2. High-Throughput Analysis of Rhizosphere Bacteria

The total bacterial community was investigated using 16S rRNA gene amplicon sequencing. Genomic DNA was extracted using the TGuide S96 Magnetic Soil/Stool DNA Kit (Tiangen Biotech, China). The V3–V4 region of the bacterial 16S rRNA gene was amplified using the primers 338F (5′–ACTCCTACGGGAGGCAGCA–3′) and 806R (5′–GGACTACHVGGGTWTCTAAT–3′). Purified amplicons were pooled and sequenced on the Illumina NovaSeq 6000 platform (2 x 250 bp paired-end reads). Raw reads were quality-filtered using Trimmomatic (v0.33) and Cutadapt (v1.9.1) [107], followed by denoising, paired-end merging, and chimera removal using DADA2 within QIIME2 to generate non-chimeric amplicon sequence variants (ASVs). Taxonomic assignment of ASVs was performed using the QIIME2 Naive Bayes classifier [108] trained on the SILVA database (v138.1) [109], supplemented by BLAST searches for consensus identification. A feature filter threshold of 2 was applied to remove rare artifacts and singletons, excluding ASVs with a total frequency under 2 reads across all samples. Sequences assigned to non-bacterial lineages, chloroplasts, or mitochondria were also filtered out prior to downstream statistical analyses. To control for varying sequencing depths across samples prior to alpha diversity calculations, feature tables were normalized via rarefaction (subsampling) to a uniform sequencing depth of 50,000 reads per sample. Rarefaction curves confirmed that sequencing depth was sufficient, reaching a clear asymptote across all sample groups.

Alpha diversity, beta diversity, and Principal Coordinates Analysis (PCoA) were performed using QIIME2 (version 2026.7), with pairwise comparisons evaluated using Student’s *t*-test (*p* < 0.05) [110]. Community dissimilarity was statistically evaluated using PERMANOVA (adonis2 function, vegan package version 2.7-6 in R) [111]. Linear discriminant analysis effect size (LEfSe) [112] was employed to identify significantly enriched microbial biomarkers between the two soil types. Functional prediction of bacterial communities was performed using FAPROTAX [113], whereas phenotypes were inferred via BugBase [114]. All bioinformatic analyses were performed using the BMK Cloud platform (Biomarker Technologies, Beijing, China).

### 4.7. Statistical Analysis

All data are reported as means ± standard deviation (SD). Prior to parametric statistical analysis, the assumptions of data normality and homogeneity of variance were verified using the Shapiro–Wilk test and Levene’s test, respectively (*p* > 0.05). Statistically significant differences were evaluated using one-way analysis of variance (ANOVA), followed by Duncan’s multiple range test (DMRT) at *p* < 0.05. Owing to the limited number of biological replicates (*n* = 3), Pearson correlation coefficients between microbial diversity indices and soil properties were calculated as a preliminary, exploratory analysis only. The statistical analysis was performed using IBM SPSS Statistics 32 for Windows (IBM Corporation, NY, USA).

## 5. Conclusions

In summary, this study provides insight into the biochemical and ecological strategies that enable *Cyanotis arachnoidea*—genetically confirmed via *rbcLa* and *trnH-psbA* barcoding—to persist in severe multi-metal-contaminated mine tailings. This plant showed notable biochemical flexibility, with enhanced anthocyanin synthesis (manifested as leaf reddening) likely mitigating heavy metal-induced oxidative stress. Proline levels decreased, whereas total phenolic and flavonoid contents and chlorophyll levels showed only minor changes. Concurrently, *C. arachnoidea* exhibited a highly specialized, element-specific metal compartmentalization strategy. It functioned effectively as a highly Zn-tolerant phytostabilizer, restricting most Zn to its roots (14.02 g kg^−1^ DW) while still maintaining substantial shoot accumulation capacity (up to 3.07 g kg^−1^ DW), and simultaneously acted as a high-translocating Cd excluder, characterized by preferential translocation of Cd to the shoots (TF > 9).

Furthermore, comprehensive characterization of the rhizosphere microbiome through HTS and culture-dependent validation revealed a marked restructuring of the microbial community associated with metal contamination. Relative abundance profiling and LEfSe cladogram analysis revealed that while metal toxicity significantly reduced overall alpha diversity, it was associated with specific microbial biomarkers and the enrichment of stress-resilient taxa such as *Sphingomonas*, *Bacillus*, Bacteroidota, and Chloroflexi. These taxonomic shifts were further substantiated by predictive microbiome profiling. FAPROTAX analysis indicated potential shifts in metabolic pathways associated with metal-stress mitigation and altered biogeochemical cycling. BugBase phenotypic prediction revealed that stress tolerance and mobile genetic elements were significantly enriched in the tailings-contaminated soil, whereas biofilm formation and potential pathogenicity were higher in the cover soil. However, in the absence of metatranscriptomic data, these predicted traits reflect only hypothetical possibilities rather than confirmed, active adaptive mechanisms.

Ultimately, the synergistic interplay between the host’s biochemical adaptations and the selective enrichment of a functionally resilient, supportive rhizosphere microbiome may underpin the survival and phytoremediation potential of *C. arachnoidea*. Further studies should focus on field-scale phytoremediation trials and the application of the isolated native metal-tolerant bacterial strains as bioinoculants to enhance plant performance and efficiency in broader remediation programs. In addition, the physiological mechanisms (particularly antioxidant enzyme activities) and molecular mechanisms driving Zn and Cd tolerance and accumulation in *C. arachnoidea* warrant investigation.

## Figures and Tables

**Table 1:** Physicochemical properties and heavy metal concentrations of untreated and autoclaved cover and tailings-contaminated soils at day 0 (before planting) and day 60 after growing *Cyanotis arachnoidea* in a pot experiment.

Properties	Day 0		Day 60			
	Cover Soil	Tailing Soil	Cover Soil	Autoclaved Cover Soil	Tailing Soil	Autoclaved Tailing Soil
Total Zn (mg kg^−1^)	1715.97 ± 10.47 ^b^	30,284.40 ± 244.16 ^e^	799.84 ± 165.94 ^a^	753.34 ± 60.13 ^a^	7953.62 ± 120.98 ^c^	10,647.83 ± 662.61 ^d^
Extractable Zn (mg kg^−1^)	3.35 ± 0.42 ^a^	153.67 ± 4.16 ^b^	30.42 ± 0.49 ^a^	20.38 ± 6.39 ^a^	596.34 ± 3.90 ^d^	335.04 ± 44.60 ^c^
Total Cd (mg kg^−1^)	101.74 ± 0.84 ^c^	865.07 ± 16.78 ^d^	5.69 ± 0.79 ^a^	3.36 ± 0.77 ^a^	32.39 ± 1.66 ^b^	26.60 ± 4.65 ^b^
Extractable Cd (mg kg^−1^)	5.12 ± 0.09 ^b^	21.87 ± 2.59 ^cd^	1.37 ± 0.02 ^a^	1.36 ± 0.24 ^a^	24.62 ± 0.87 ^d^	20.88 ± 2.69 ^c^
Total Fe (mg kg^−1^)	16,549.07 ± 21.67 ^e^	24,897.90 ± 60.69 ^f^	8920.10 ± 29.85 ^b^	5266.69 ± 234.88 ^a^	10,256.82 ± 323.50 ^c^	14,148.56 ± 32.56 ^d^
pH	7.54 ± 0.47 ^b^	6.62 ± 0.28 ^a^	6.78 ± 0.05 ^a^	6.82 ± 0.22 ^a^	6.53 ± 0.34 ^a^	6.66 ± 0.03 ^a^
Organic C (%)	2.14 ± 0.07 ^a^	4.67 ± 0.30 ^b^	11.01 ± 0.19 ^d^	8.74 ± 0.35 ^c^	12.63 ± 0.62 ^e^	7.77 ± 1.98 ^c^
Organic matter (%)	3.69 ± 0.12 ^a^	8.48 ± 0.39 ^b^	18.99 ± 0.33 ^d^	15.07 ± 0.61 ^c^	21.77 ± 1.07 ^e^	13.40 ± 3.41 ^c^
Total N (%)	0.06 ± 0.01 ^a^	0.23 ± 0.01 ^e^	0.15 ± 0.01 ^c^	0.11 ± 0.01 ^b^	0.24 ± 0.01 ^e^	0.22 ± 0.01 ^d^
C:N ratio	33.91 ± 2.31 ^b^	20.28 ± 1.30 ^a^	75.15 ± 2.29 ^d^	77.50 ± 5.21 ^d^	52.61 ± 2.59 ^c^	36.03 ± 8.96 ^b^
Available P (mg kg^−1^)	354.29 ± 5.11 ^d^	727.37 ± 13.58 ^e^	264.53 ± 4.37 ^c^	207.97 ± 11.61 ^b^	33.61 ± 0.78 ^a^	24.79 ± 2.04 ^a^
Available K (mg kg^−1^)	1742.85 ± 36.16 ^c^	7611.35 ± 60.43 ^d^	165.03 ± 11.85 ^b^	106.36 ± 14.34 ^a^	85.59 ± 6.65 ^a^	90.55 ± 5.14 ^a^

Note: Data are expressed on a dry weight soil basis and presented as means ± SD (*n* = 3). Different superscript letters within the same row indicate significant differences (*p* < 0.05).

**Table 2:** Total viable bacterial counts (log CFU g^−1^ soil) in bulk soil at day 0 and rhizosphere soil at day 60, alongside culture-dependent diversity indices of bacteria isolated from the rhizosphere of *Cyanotis arachnoidea* under different soil treatments.

Soil Treatment	Number of Bacteria in Bulk Soil (Day 0) (log CFU g^−1^ Soil)	Rhizosphere Soil			
		Number (log CFU g^−1^ Soil)	Shannon-Wiener Index (*H*)	Richness (*S*)	Evenness (*E_H_*)
Cover soil	5.37 ± 0.09 ^c^	5.57 ± 0.04 ^c^	1.68 ± 0.17 ^bc^	8.33 ± 1.15 ^b^	0.79 ± 0.04 ^a^
Autoclaved cover soil	4.55 ± 0.07 ^a^	5.08 ± 0.10 ^a^	1.76 ± 0.06 ^c^	6.67 ± 0.58 ^ab^	0.94 ± 0.03 ^a^
Tailing soil	5.14 ± 0.12 ^b^	5.35 ± 0.03 ^b^	1.47 ± 0.16 ^b^	6.67 ± 0.58 ^ab^	0.78 ± 0.12 ^a^
Autoclaved tailing soil	4.52 ± 0.08 ^a^	5.38 ± 0.04 ^b^	1.21 ± 0.08 ^a^	5.00 ± 1.00 ^a^	0.77 ± 0.14 ^a^

Note: Data are means ± SD (*n* = 3). Different superscript letters within the same column indicate significant differences (*p* < 0.05).

## Data Availability

The nucleotide sequences generated in this study have been deposited in the GenBank database, and the accession numbers are provided in Section 4.

## Supplementary Materials

The following supporting information can be downloaded at: https://www.mdpi.com/article/10.3390/ijms27219684/s1.
