# Supplementary material for: Ecological Responses of Cyanotis arachnoidea C.B. Clarke and Its Rhizosphere Microbiome in Zinc Mine Tailings: Insights for Post-Mining Restoration
Source: Int J Mol Sci. 2026 Oct 29;27(21):9684. doi: 10.3390/ijms27219684 (PMC13762631; doi:10.3390/ijms27219684)
Supplement: Supplementary file 1 [file ijms-27-09684-s001.zip › ijms-4508421-supplementary.pdf]

Table S1. Morphological and molecular identification of culturable bacterial isolates obtained from the rhizosphere of *Cyanotis arachnoidea* in cover soil and tailings-contaminated soil.

Isolate code	GenBank accession no.	Suggestion of GenBank at the species level	Similarity (%)	Colony morphology						Gram stain	Endospore	EPS
				Shape	Margin	Elevation	Surface	Opacity	Color			
CSCT01	PX957012	*Staphylococcus* sp. GR 5-08	99.93	Circular	Entire	Flat	Smooth	Opaque	Creamy-yellow	Positive	-	-
CSCT02	PX957282	*Pseudomonas machongensis* MH2	99.93	Circular	Undulate	Flat	Rough	Translucent	Creamy-yellow	Negative	-	+
CSCT04	PX956988	*Priestia aryabhattai* LT203	99.72	Irregular	Undulate	Raised	Dull	Opaque	Creamy-yellow	Positive	+	+
CSCT07	PX957011	*Rhizobium ipomoeae* SS2	99.48	Circular	Entire	Raised	Glistening	Opaque	Creamy-yellow	Negative	-	+
CSCT08	PX957013	*Staphylococcus* sp. GR 5-08	99.86	Circular	Entire	Raised	Smooth	Opaque	Cream	Positive	-	-
CSCT09	PX956988	*Fictibacillus barbaricus* NIOT-Ba-23	99.30	Circular	Entire	Flat	Smooth	Opaque	Creamy-yellow	Positive	+	+
CSCT10	PX956801	*Fictibacillus phosphorivorans* V6608	99.72	Circular	Entire	Raised	Smooth	Opaque	Creamy-yellow	Positive	+	+
TSCT01	PX956829	*Krasilnikoviella flava* 126 LSS	99.85	Circular	Entire	Raised	Smooth	Opaque	Creamy-yellow	Positive	+	+
TSCT02	PX956988	*Priestia aryabhattai* LT203	99.93	Irregular	Undulate	Raised	Rough	Opaque	Creamy-yellow	Positive	+	+
TSCT03	PX956716	*Arthrobacter phenanthrenivorans* IHB B15110	98.94	Circular	Entire	Raised	Smooth	Opaque	Creamy-white	Positive	-	+
TSCT05	PX956838	*Nocardia abscessus* SM 545	99.71	Circular	Entire	Raised	Dull	Opaque	White	Positive	-	+
TSCT08	PX956990	*Pseudarthrobacter oxydans* FT121	99.57	Circular	Entire	Raised	Smooth	Opaque	Creamy-white	Positive	-	+
TSCT09	PX956946	*Pontibacter cellulosilyticus* SD6	97.31	Circular	Entire	Raised	Smooth	Smooth	Orange	Negative	-	+
TSCT10	PX95694	*Nocardioides deserti* SC8A-24	98.34	Circular	Entire	Raised	Smooth	Opaque	Cream	Positive	-	+

Note: (+), detected; (-), not detected; EPS, exopolysaccharide production.

Table S2. Preliminary Pearson correlation coefficients (*r*) between soil physicochemical properties, heavy metal concentrations, and bacterial alpha diversity indices in the rhizosphere of *Cyanotis arachnoidea.*

Index	Total Zn	Ext. Zn	Total Cd	Ext. Cd	Total Fe	pH	C/N ratio	Available P	Available K
Chao1	-0.990**	-0.980**	-0.975**	-0.988**	-0.985**	0.492	0.989**	0.985**	0.981**
Shannon	-0.995**	-0.994**	-0.991**	-0.993**	-0.970**	0.551	0.994**	0.993**	0.989**
PD whole tree	0.436	0.477	0.411	0.459	0.456	0.201	-0.492	-0.453	-0.543

** Correlation is significant at the 0.01 level (2-tailed).

* Correlation is significant at the 0.05 level (2-tailed).
